# Diagnostic performance of DCE-MRI radiomics in predicting axillary lymph node metastasis in breast cancer patients: A meta-analysis

**DOI:** 10.1371/journal.pone.0314653

**Published:** 2024-12-03

**Authors:** Fei Dong, Jie Li, Junbo Wang, Xiaohui Yang

**Affiliations:** 1 Department of Medical Imaging, Yuncheng Central Hospital Affiliated to Shanxi Medical University, Yuncheng, Shanxi Province, China; 2 Department of Anesthesiology, Yuncheng Central Hospital Affiliated to Shanxi Medical University, Yuncheng, Shanxi Province, China; Tabriz University of Medical Sciences, ISLAMIC REPUBLIC OF IRAN

## Abstract

Radiomics offers a novel strategy for the differential diagnosis, prognosis evaluation, and prediction of treatment responses in breast cancer. Studies have explored radiomic signatures from dynamic contrast-enhanced magnetic resonance imaging (DCE-MRI) for predicting axillary lymph node metastasis (ALNM) and sentinel lymph node metastasis (SLNM), but the diagnostic accuracy varies widely. To evaluate this performance, we conducted a meta-analysis performing a comprehensive literature search across databases including PubMed, EMBASE, SCOPUS, Web of Science (WOS), Cochrane Library, China National Knowledge Infrastructure (CNKI), Wanfang Data, and the Chinese BioMedical Literature Database (CBM) until March 31, 2024. The pooled sensitivity, specificity, positive likelihood ratio (PLR), negative likelihood ratio (NLR), diagnostic odds ratio (DOR), and the area under the receiver operating characteristic curve (AUC) were calculated. Twenty-four eligible studies encompassing 5588 breast cancer patients were included in the meta-analysis. The meta-analysis yielded a pooled sensitivity of 0.81 (95% confidence interval [CI]: 0.77–0.84), specificity of 0.85 (95%CI: 0.81–0.87), PLR of 5.24 (95%CI: 4.32–6.34), NLR of 0.23 (95%CI: 0.19–0.27), DOR of 23.16 (95%CI: 17.20–31.19), and AUC of 0.90 (95%CI: 0.87–0.92), indicating good diagnostic performance. Significant heterogeneity was observed in analyses of sensitivity (I^2^ = 74.64%) and specificity (I^2^ = 83.18%). Spearman’s correlation coefficient suggested no significant threshold effect (P = 0.538). Meta-regression and subgroup analyses identified several potential heterogeneity sources, including data source, integration of clinical factors and peritumor features, MRI equipment, magnetic field strength, lesion segmentation, and modeling methods. In conclusion, DCE-MRI radiomic models exhibit good diagnostic performance in predicting ALNM and SLNM in breast cancer. This non-invasive and effective tool holds potential for the preoperative diagnosis of lymph node metastasis in breast cancer patients.

## Introduction

Breast cancer is one of the most common malignant tumors and a leading cause of cancer-related deaths mortality among women worldwide [1]. The status of axillary lymph node (ALN) is crucial for tumor staging, treatment decisions, and prognosis [2, 3]. Patients with axillary lymph node metastasis (ALNM) face a higher risk of distant metastasis and lower overall survival compared to those without node metastasis [4]. Standard methods for confirming ALN status, such as sentinel lymph node biopsy (SLNB) and axillary lymph node dissection (ALND), are invasive and carry risks like shoulder dysfunction, lymphedema, subcutaneous effusion, and nerve injury [5, 6]. Additionally the significant false-negative rate of SLNB complicates [7], highlighting the need for non-invasive and accurate diagnostic methods.

Dynamic contrast-enhanced magnetic resonance imaging (DCE-MRI) offers detailed morphological and hemodynamic information about tumor lesions, making it a valuable tool in breast cancer staging [8]. Despite its potential pre-operative ALN assessment [9, 10], DCE-MRI’s diagnostic performance for ALNM is suboptimal. Evaluation based on qualitative or semi-qualitative features can be influenced by the radiologist’s expertise and experience [11]. Radiomics, which extracts high-throughput quantitative features from medical images within the region of interest (ROI), offers a novel and objective approach for comprehensive and detailed tumor heterogeneity assessment [12]. Radiomics has shown great potential in diagnosis, prognosis evaluation, and predicting therapeutic response by analyzing the relationships between various radiomic features and clinical information [13, 14].

Recently, interest has surged in applying DCE-MRI radiomics for assessing ALN status in breast cancer, with numerous models developed and validated for predicting ALNM and sentinel lymph node metastasis (SLNM) [9, 15–17]. However, inconsistencies across studies arise from variations in sample size, MRI protocols, radiomic features, and model construction. To address these disparities, we conducted a meta-analysis to systematically evaluate the diagnostic performance of DCE-MRI radiomics in predicting ALNM and SLNM in breast cancer.

## Materials and methods

### Literature search and selection

This meta-analysis adhered to the Preferred Reporting Items for Systematic Reviews and Meta-Analyses (PRISMA) guideline (S1 Checklist) [18]. Comprehensive searches were conducted in databases including PubMed, EMBASE, SCOPUS, Web of Science (WOS), Cochrane Library, China National Knowledge Infrastructure (CNKI), Wanfang Data, and the Chinese BioMedical Literature Database (CBM) for relevant studies published from up to March 31, 2024. The search utilized the following MeSH terms and variants: (‘Lymphatic Metastasis’[Mesh] OR ‘Lymph Nodes’[Mesh] OR ‘lymph’[Title/Abstract]) AND (‘Breast Neoplasms’[Mesh] OR ‘breast cancer’[Title/Abstract] OR ‘breast carcinoma’[Title/Abstract]) AND (‘Magnetic Resonance Imaging’[Mesh] OR ‘magnetic resonance imaging’[Title/Abstract] OR ‘MRI’[Title/Abstract]) AND (‘Radiomics’[Mesh] OR ‘radiomic*’[Title/Abstract]). Searches were restricted to English and Chinese articles. Additionally, references of relevant articles were manually reviewed to identify further eligible studies.

The inclusion criteria included: (1) studies involving breast cancer patients with definitive pathological outcomes for ALNM or SLNM; (2) studies establishing models to classify ALNM or SLNM based on DCE-MRI radiomics; (3) studies providing sufficient data for constructing a 2 × 2 contingency table to calculate sensitivity and specificity. Exclusions criteria included: (1) overlapping data; (2) incomplete or missing analytical data for constructing the 2 × 2 contingency table; (3) radiomics of MRI sequences other than DCE-MRI; (4) studies focusing on predicting ALN burden. Case reports, reviews, conference abstracts, and animal studies were also excluded. Two independent authors (JL, JW) performed the literature search and selection, and resolved any disagreements through discussion.

### Data extraction

Two authors (FD, JL) independently extracted the following data from each study: first author, publication year, country/region, study design, sample size, data source, reference standard, MRI equipment, magnetic field strength, DCE phase, ROI, clinical factors, peritumoral features, and feature selection and model construction details. True positive (TP), false positive (FP), false negative (FN), and true negative (TN) values were also extracted to construct the 2 × 2 contingency table. Any conflicts were resolved through discussion.

### Methodological quality assessment

Methodological quality was assessed using the Quality Assessment of Diagnostic Accuracy Studies (QUADAS-2) tool [19]. Bias in patient selection, index test, reference standard, and flow and timing was rated as high, unclear, or low risk. Applicability concerns in patient selection, index test, and reference standard were rated as high, unclear, or low. In addition, the METhodological RadiomICs Score (METRICS), a scoring tool for quality assessment of radiomics research, was used to assess the methodological quality of included studies [20]. The quality was categorized according to METRICS score as very low (0 ≤ score < 20%), low (20 ≤ score < 40%), moderate (40 ≤ score < 60%), good (60 ≤ score < 80%), and excellent (80 ≤ score ≤ 100%). Two authors (FD, JL) independently assessed the methodological quality, and resolved conflicts through discussion.

### Statistical analysis

Predictive accuracy of DCE-MRI radiomic model was assessed by calculating pooled sensitivity, specificity, positive likelihood ratio (PLR), negative likelihood ratio (NLR), and diagnostic odds ratio (DOR) with corresponding 95% confidence interval (95%CI). The summary receiver operating characteristic (SROC) curve was plotted. The area under the curve (AUC) was estimated to summarize diagnostic performance, with discrimination ability categorized as excellent (AUC>0.90), good (0.80–0.90), fair (0.70–0.80), poor (0.60–0.70), and unqualified (0.50–0.60). Heterogeneity among studies was assessed using the I^2^ statistic with Cochrane Q test, with I^2^ >50% and P<0.05 indicating significant heterogeneity, warranting a random-effect model for pooled analysis. In cases of significant heterogeneity, Spearman’s correlation coefficient was calculated to assess the threshold effect. Meta-regression and subgroup analyses were conducted to explore heterogeneity sources, considering covariates such as region, data source, outcome, combining clinical factors, combining peritumoral features, MRI equipment, magnetic field strength, ROI, lesion segmentation method, and classifier type. We used Leave-One-Out sensitivity analysis to test result robustness and Deek’s funnel plot to assess publication bias. Clinical utility of DCE-MRI radiomics was evaluated using the Fagan plot, calculating posttest probability of lymph node metastasis based on pretest probability. All analyses were conducted using STATA 16.0 (SataCorp, TX, USA). P value <0.05 was considered statistically significant.

## Results

### Baseline characteristics of studies included in meta-analysis

**Fig 1:**
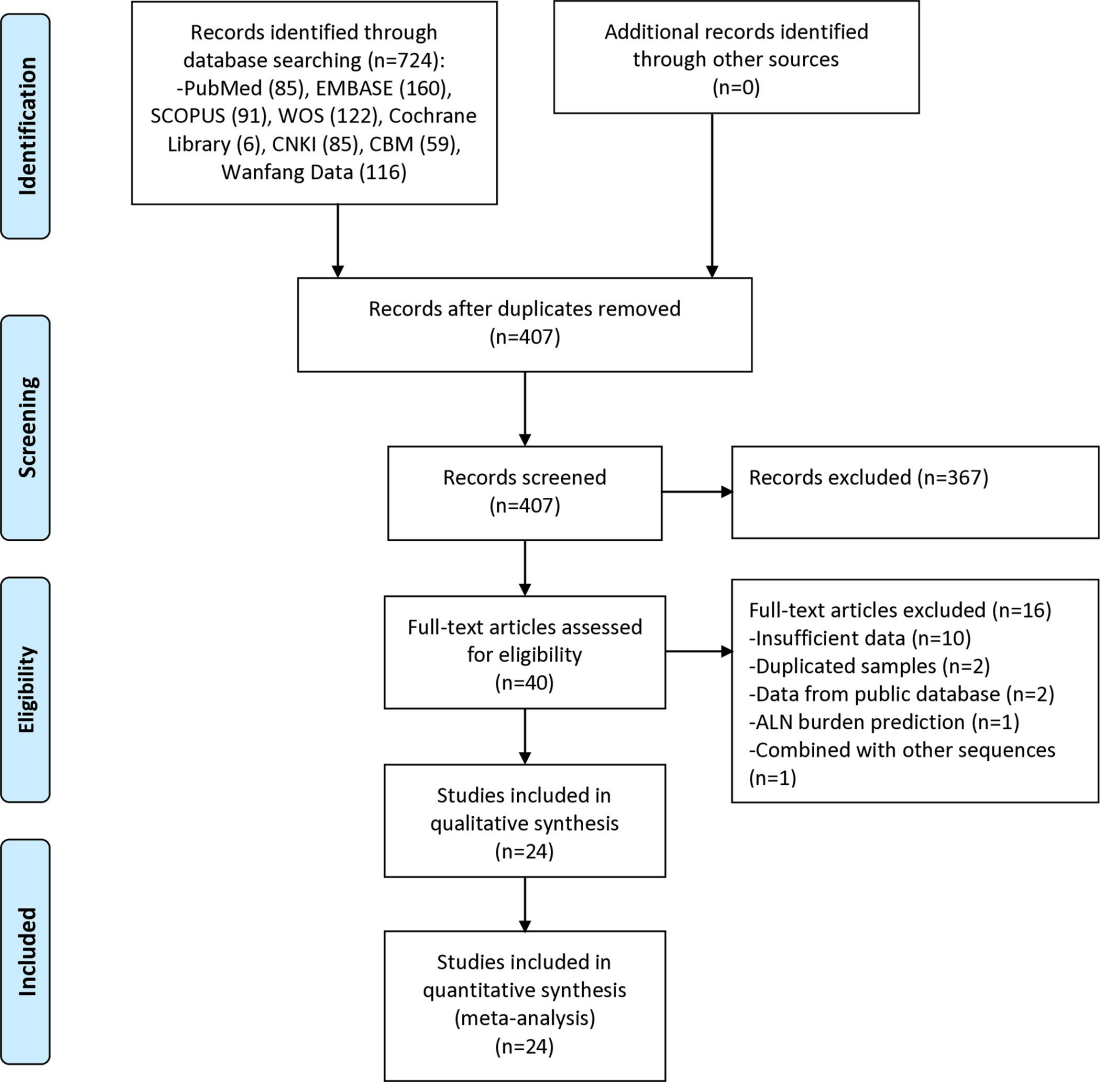
Flow diagram of literature search and selection.

The literature search initially yielded 724 records, of which 407 unique articles remained after duplicate removal (Fig 1) Title and abstract screening led to the exclusion of 367 items. A total of 40 articles were selected for full-text review, with 16 ultimately excluded for various reasons. All the identified articles with eligibility status are listed in S1 Table. In total, 24 eligible studies comprising 5588 breast cancer patients were included in the meta-analysis [9, 10, 15–17, 21–39].

Among these studies, four were published in Chinese [36–39], while the remainder were in English. Except for two studies conducted outside China [15, 17], all were retrospective studies performed in China. Most studies were single-center; however, three recruited patients from two institutions [10, 24, 35]. Six studies developed models for SLNM prediction, while the others focused on ALNM. Ten studies incorporated combined clinical factors into radiomic-based models, and seven combined intratumoral and peritumoral features. The ROI was the breast tumor in 21 studies, the lymph node in two [24, 31], and both the tumor and lymph node in one [35]. The DCE phases analyzed included the strongest enhanced phase, first postcontrast phase (CE1), second postcontrast phase (CE2), and third postcontrast phase (CE3) in 7, 3, 9, and 5 studies, respectively. Nineteen studies manually segmented the ROI, while five studies used semi-automatic or automatic segmentation [15, 17, 20, 31, 32]. Logistic regression (LR) and support vector machine (SVM) were the most commonly used classifiers, featured in 11 and 9 studies, respectively. The baseline characteristics of all studies are summarized in Table 1, while details of MRI scanning and model construction are presented listed in Table 2. The extracted analytical data and corresponding 2 × 2 contingency tables showing TP, FP, FN, and TN for each study are presented in S2 Table.

**Table 1:** Baseline characteristics of studies included in the meta-analysis.

Study	Design	Region	Sample size	Outcome	Reference standard	Data source	Clinical information	Peritumoral features
Arefan D, 2020	Retro	USA	154	ALNM	Surgical resection or FNA	Single center	No	No
Chen DX, 2022	Retro	China	243	ALNM	Surgical resection	Single center	No	No
Chen JM, 2021	Retro	China	140	ALNM	Surgical resection or FNA	Single center	No	No
Chen WY, 2024	Retro	China	410	ALNM	Surgical resection	Single center	Yes	No
Cheng Y, 2022	Retro	China	208	SLNM	Surgical resection	Single center	No	Yes
Cui XY, 2019	Retro	China	115	ALNM	Surgical resection	Single center	No	No
Han L, 2019	Retro	China	411	ALNM	Surgical resection	Single center	Yes	No
Liu CL, 2019	Retro	China	163	SLNM	Surgical resection	Single center	Yes	Yes
Liu J, 2019	Retro	China	62	SLNM	Surgical resection	Single center	No	No
Liu Y, 2022	Retro	China	312	ALNM	Surgical resection	Single center	No	Yes
Ma MM, 2022	Retro	China	42	SLNM	Surgical resection	Single center	No	No
Santucci D, 2021	Retro	Italy	99	ALNM	Surgical resection	Single center	No	No
Shan YN, 2020	Retro	China	145	ALNM	Surgical resection	Two centers	No	No
Song DL, 2022	Retro	China	432	ALNM	Surgical resection	Single center	Yes	No
Tang YQ, 2022	Retro	China	337	ALNM	Surgical resection	Single center	Yes	No
Wang CH, 2021	Retro	China	186	SLNM	Surgical resection	Single center	No	No
Wang Q, 2024	Retro	China	485	ALNM	Surgical resection	Two centers	No	No
Wang YX, 2024	Retro	China	473	ALNM	Surgical resection or FNA	Single center	Yes	Yes
Zhan CA, 2021	Retro	China	166	ALNM	Surgical resection	Single center	No	Yes
Zhang CM, 2023	Retro	China	215	ALNM	Surgical resection	Single center	No	Yes
Zhang JW, 2023	Retro	China	263	ALNM	Surgical resection	Two centers	Yes	No
Zhao NN, 2023	Retro	China	180	ALNM	Surgical resection	Single center	Yes	Yes
Zhu YD, 2021	Retro	China	177	SLNM	Surgical resection	Single center	Yes	No
Zhu YQ, 2022	Retro	China	169	ALNM	Surgical resection	Single center	Yes	No

ALNM: axillary lymph node metastasis; FNA: fine needle aspiration; Retro: retrospective; SLNM: sentinel lymph node metastasis.

**Table 2:** Features of MRI scanning and prediction model construction.

Study	Equipment	Phase	ROI	Lesion segmentation	Feature selection	Classifier
Arefan D, 2020	Siemens (3.0T)	CE2	Tumor	Semi-automatic	LASSO	LDA
Chen DX, 2022	GE (3.0T)	CE3	Tumor	Manual	Pearson correlation analysis, LASSO	LR
Chen JM, 2021	GE (3.0T)	The strongest enhanced phase	Tumor	Manual	mRMR, LASSO	LR
Chen WY, 2024	Siemens (1.5T)	CE2	Tumor	Manual	SelectKBest, LASSO	LR
Cheng Y, 2022	GE (1.5T)	The strongest enhanced phase	Tumor	Manual	LASSO	LR
Cui XY, 2019	Siemens (3.0T)	CE2	Tumor	Semi-automatic	LASSO	SVM
Han L, 2019	GE (1.5T)	CE1	Tumor	Manual	LASSO, LOOCV	SVM
Liu CL, 2019	GE (1.5T)	CE1	Tumor	Manual	Spearman correlation analysis, LASSO	LR
Liu J, 2019	GE (3.0T)	The strongest enhanced phase	Tumor	Manual	SelectKBest, LASSO	SVM
Liu Y, 2022	Siemens (3.0T)	CE2	Tumor	Manual	Spearman correlation analysis, LASSO	SVM
Ma MM, 2022	GE (3.0T)	CE3	Lymph node	Automatic	ANOVA	SVM
Santucci D, 2021	GE (3.0T)	CE2	Tumor	Semi-automatic	RF	RF
Shan YN, 2020	Siemens (3.0T)	CE1	Lymph node	Manual	ANOVA, Spearman correlation analysis, LASSO	LR
Song DL, 2022	Philips (3.0T)	CE2	Tumor	Semi-automatically	LASSO	LR
Tang YQ, 2022	Siemens (3.0T)	The strongest enhanced phase	Tumor	Manual	LASSO	SVM
Wang CH, 2021	Siemens (3.0T)	The strongest enhanced phase	Tumor	Manual	mRMR, LASSO	LR
Wang Q, 2024	Siemens (1.5T, 3.0T), Philips (3.0T)	CE2	Tumor	Manual	LASSO	RF
Wang YX, 2024	GE (3.0T)	CE3	Tumor	Manual	SelectKBest, LASSO	LR
Zhan CA, 2021	Siemens (3.0T)	The strongest enhanced phase	Tumor	Manual	Spearman correlation analysis, SVM	SVM
Zhang CM, 2023	GE (3.0T)	CE3	Tumor	Manual	Pearson correlation analysis, mRMR, LASSO	RF
Zhang JW, 2023	GE (3.0T)	The strongest enhanced phase	Tumor + lymph node	Manual	SelectKBest, LASSO	LR
Zhao NN, 2023	Philips (3.0T)	CE2	Tumor	Manual	SelectKBest, LASSO	SVM
Zhu YD, 2021	GE (3.0T)	CE2	Tumor	Manual	LASSO	SVM
Zhu YQ, 2022	GE (1.5T)	CE3	Tumor	Manual	LASSO	LR

ANOVA: analysis of variance; CE1/CE2/CE3: the first/second/third postcontrast phase; LASSO: least absolute shrinkage and selection operator; LDA: liner discriminant analysis; LOOCV: leave-one-out cross-validation; LR: logistic regression; MRI: magnetic resonance image; mRMR: minimal redundancy maximum relevancy; RF: random forest; ROI: region of interest; SVM: support vector machine.

### Methodological quality assessment

**Fig 2:**
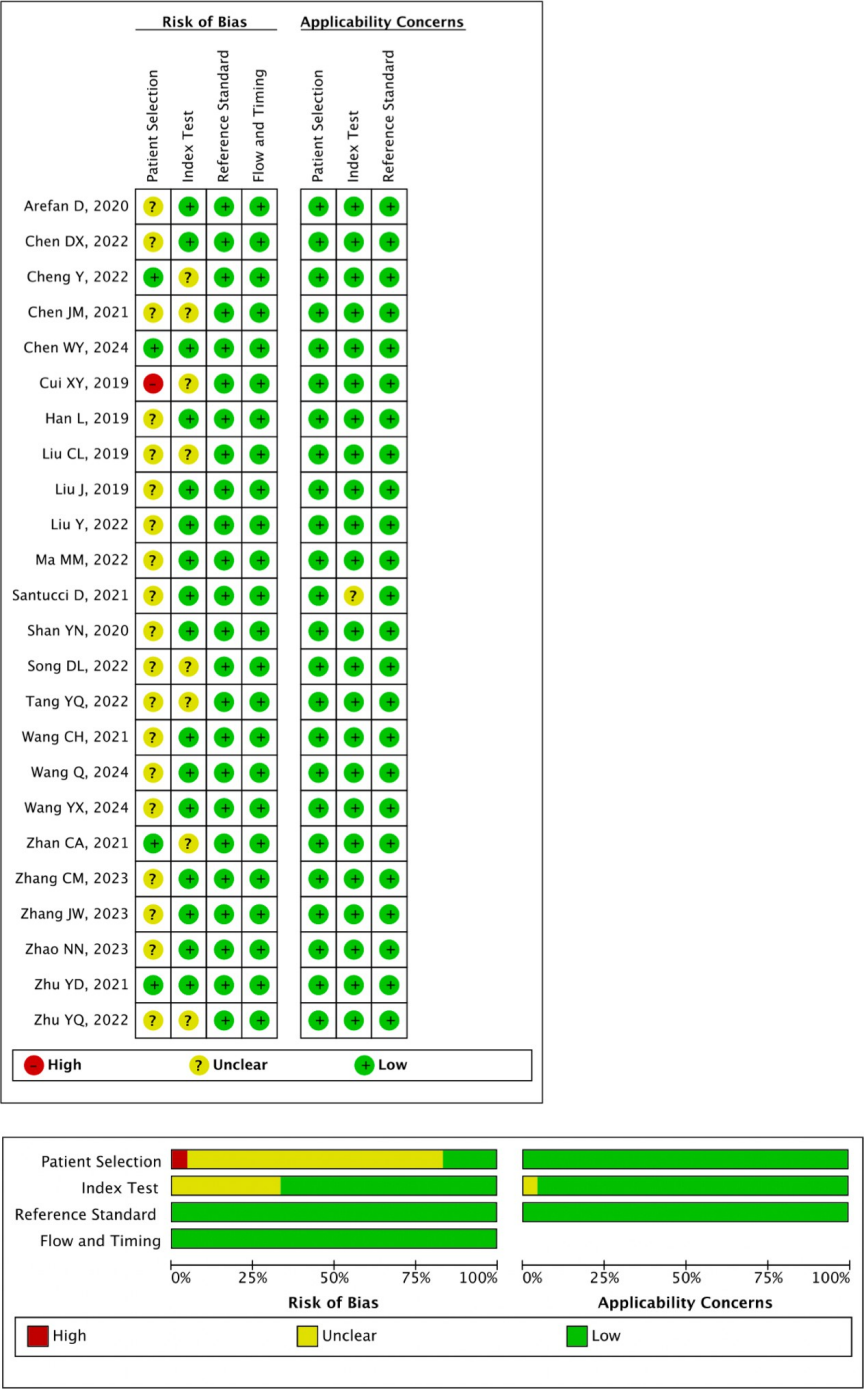
Methodological quality assessment of the included studies using the Quality Assessment of Diagnostic Accuracy Studies (QUADAS-2) tool.

In the QUADAS-2 assessment, the majority of studies exhibited unclear risk of bias in the patient selection domain due to insufficient descriptions of the patient selection process. One study showed high risk of bias in this domain [20], while four studies demonstrated low risk of bias [16, 26, 27, 29]. Regarding the index test, eight studies were considered to have unclear risk of bias due to the unknown use of a blinded setting [16, 20, 22, 26, 32, 33, 36, 39]. The remaining studies were rated with low risk of bias. Details of the risk of bias and applicability concerns are illustrated in Fig 2.

The METRICS assessment of included studies is detailed in S3 Table, with a median score of 69.0% (range: 53.1–90.3%). Three studies were categorized as moderate quality [17, 21, 23], 2 as excellent quality [10, 35], and the others as good quality.

### Pooled analysis

**Fig 3:**
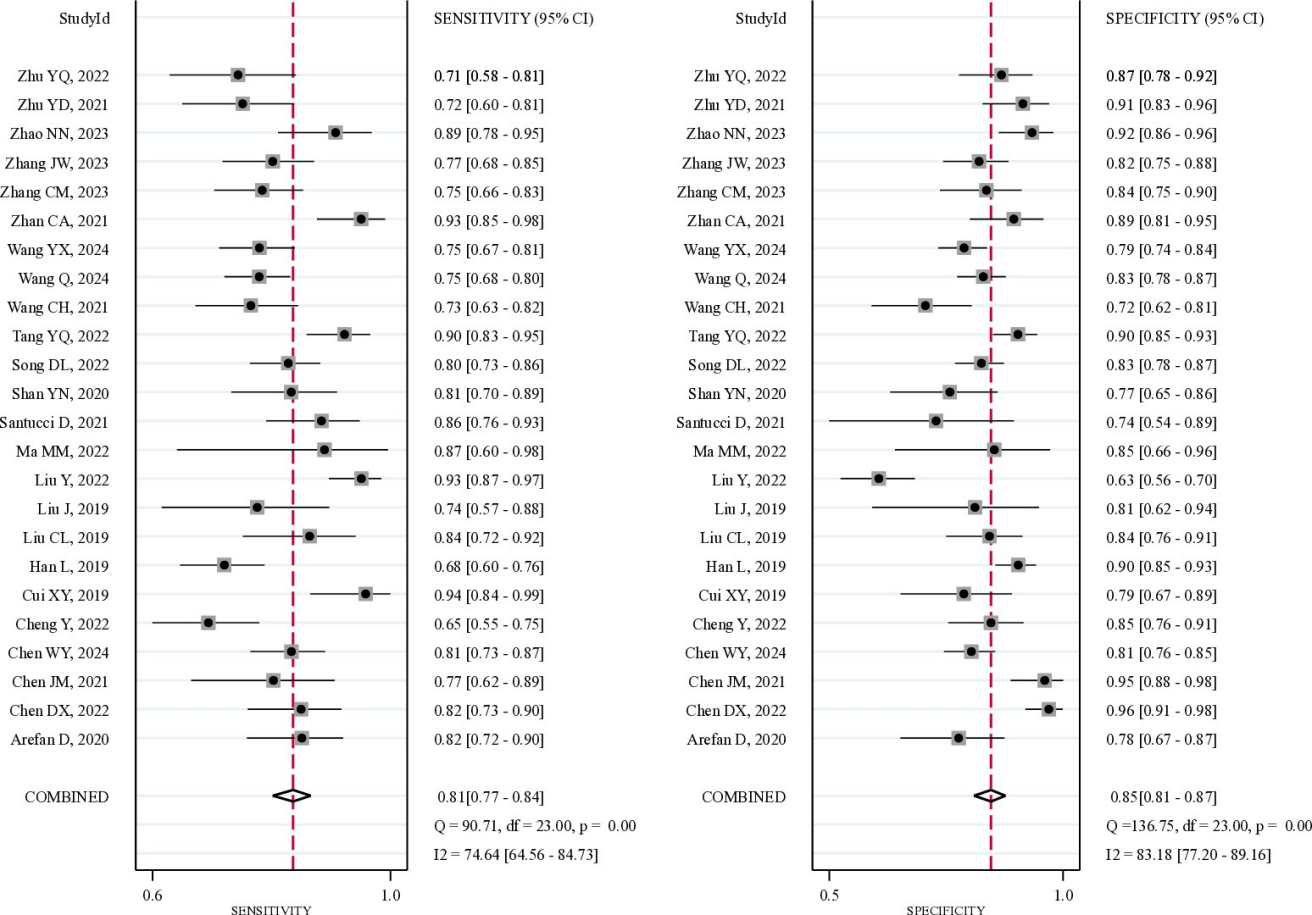
Forest plots of sensitivity and specificity of DCE-MRI radiomics in predicting lymph node metastasis in breast cancer.

**Fig 4:**
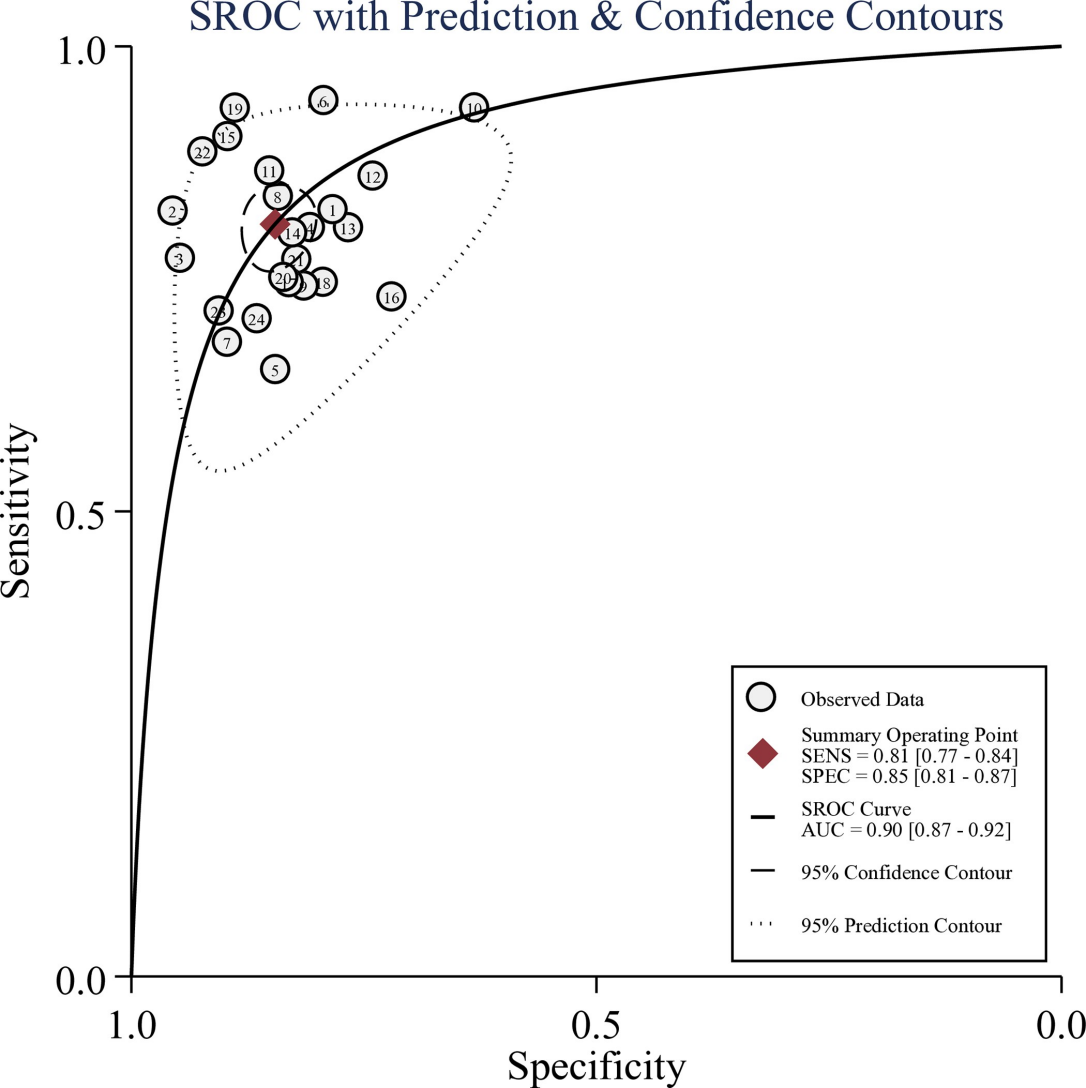
The summary receiver operating characteristics curve of the diagnostic performance of DCE-MRI radiomics in predicting lymph node metastasis in breast cancer.

As illustrated in Fig 3, the meta-analysis of 24 studies revealed that the pooled sensitivity and specificity for DCE-MRI radiomic signatures for predicting ALNM and SLNM in breast cancer patients were 0.81 (95%CI: 0.77–0.84) and 0.85 (95%CI: 0.81–0.87), respectively. The pooled analysis produced a PLR of 5.24 (95%CI: 4.32–6.34), an NLR of 0.23 (95%CI: 0.19–0.27), and a DOR of 23.16 (95%CI: 17.20–31.19). The SROC curve was plotted in Fig 4, yielding an AUC of 0.90 (95%CI: 0.87–0.92). Therefore, DCE-MRI radiomic signatures exhibited a good predictive ability for assessing the risk of ALNM and SLNM in breast cancer patients.

### Heterogeneity

Significant heterogeneity was observed in the pooled analysis of sensitivity (I^2^ = 74.64%) and specificity (I^2^ = 83.18%).The Spearman’s correlation coefficient indicated no evidence of a threshold effect (coefficient = -0.131, P = 0.538). Univariate meta-regression analysis and subgroup analyses identified various factors contributing to heterogeneity, including data source, outcome assessment, clinical factors, peritumor features, MRI equipment, magnetic field, lesion segmentation, and classifier type (Table 3).

**Table 3:** Results of univariate meta-regression and subgroup analyses.

Subgroup	No. of studies	Sensitivity (95%CI)	P1	Specificity (95%CI)	P2
Region			<0.01		0.32
China	22	0.81 (0.77–0.84)		0.85 (0.82–0.88)	
Outside China	2	0.85 (0.75–0.95)		0.77 (0.62–0.92)	
Data source			0.02		<0.01
Single center	21	0.81 (0.78–0.85)		0.85 (0.82–0.88)	
Two centers	3	0.78 (0.67–0.88)		0.81 (0.72–0.90)	
Outcome			<0.01		<0.01
SLNM	6	0.75 (0.67–0.83)		0.84 (0.77–0.90)	
ALNM	18	0.82 (0.79–0.86)		0.85 (0.81–0.88)	
Combining clinical factors			<0.001		<0.001
Yes	10	0.79 (0.74–0.85)		0.86 (0.82–0.90)	
No	14	0.82 (0.78–0.87)		0.83 (0.79–0.87)	
Combining peritumoral features			<0.001		<0.001
Yes	7	0.84 (0.75–0.90)		0.83 (0.76–0.88)	
No	17	0.79 (0.76–0.83)		0.85 (0.81–0.88)	
MRI equipment			<0.001		<0.001
Siemens	8	0.87 (0.83–0.90)		0.80 (0.74–0.85)	
GE	13	0.76 (0.72–0.81)		0.87 (0.84–0.90)	
Magnetic field			<0.01		<0.001
1.5T	5	0.74 (0.66–0.82)		0.86 (0.80–0.92)	
3.0T	18	0.83 (0.79–0.86)		0.84 (0.81–0.88)	
ROI			0.08		0.18
Tumor	21	0.81 (0.77–0.85)		0.85 (0.82–0.88)	
Lymph node	2	0.83 (0.70–0.96)		0.81 (0.67–0.94)	
Lesion segmentation			<0.001		<0.01
Manual	19	0.80 (0.76–0.83)		0.85 (0.82–0.88)	
Semi-automatic or automatic	5	0.86 (0.80–0.92)		0.80 (0.72–0.89)	
Classifier			<0.01		<0.001
LR	11	0.77 (0.72–0.83)		0.85 (0.80–0.89)	
SVM	9	0.87 (0.79–0.92)		0.86 (0.80–0.91)	

ALNM: axillary lymph node metastasis; LR: logistic regression; ROI: region of interest; SLNM: sentinel lymph node metastasis; SVM: support vector machine.

Higher pooled sensitivity and specificity were observed in studies using single-center data compared to those using two-center data (sensitivity: 0.81 *vs* 0.78; specificity: 0.85 *vs* 0.81). Studies focused on ALNM exhibited greater sensitivity and specificity than those on SLNM (sensitivity: 0.82 *vs* 0.78; specificity: 0.85 *vs* 0.84), Additionally, studies adopting SVM for prediction models showed improved sensitivity and specificity compared to those using LR (sensitivity: 0.87 *vs* 0.77; specificity: 0.86 *vs* 0.85). Models than incorporated clinical factors displayed higher specificity (0.86 *vs* 0.83) but lower sensitivity (0.79 *vs* 0.82) compared to radiomic models alone. Furthermore, models integrating both intratumoral and peritumor features demonstrated higher sensitivity (0.84 *vs* 0.79) and but slightly lower specificity (0.83 vs 0.85) than those using only intratumoral features. 3.0T imaging had higher sensitivity (0.83 *vs* 0.74) but slightly reduced specificity (0.84 *vs* 0.86) compared to 1.5T imaging. Siemens MRI equipment was associated with higher sensitivity (0.87 *vs* 0.76) but lower specificity (0.80 *vs* 0.87) than GE equipment. Semi-automatic or automatic segmentation improved sensitivity (0.86 *vs* 0.80) but lowered the specificity (0.80 *vs* 0.85) compared to manual segmentation. No significant differences in pooled sensitivity and specificity were observed between studies using only breast tumors as the ROI and those using lymph nodes (P>0.05).

### Sensitivity analysis and publication bias

**Fig 5:**
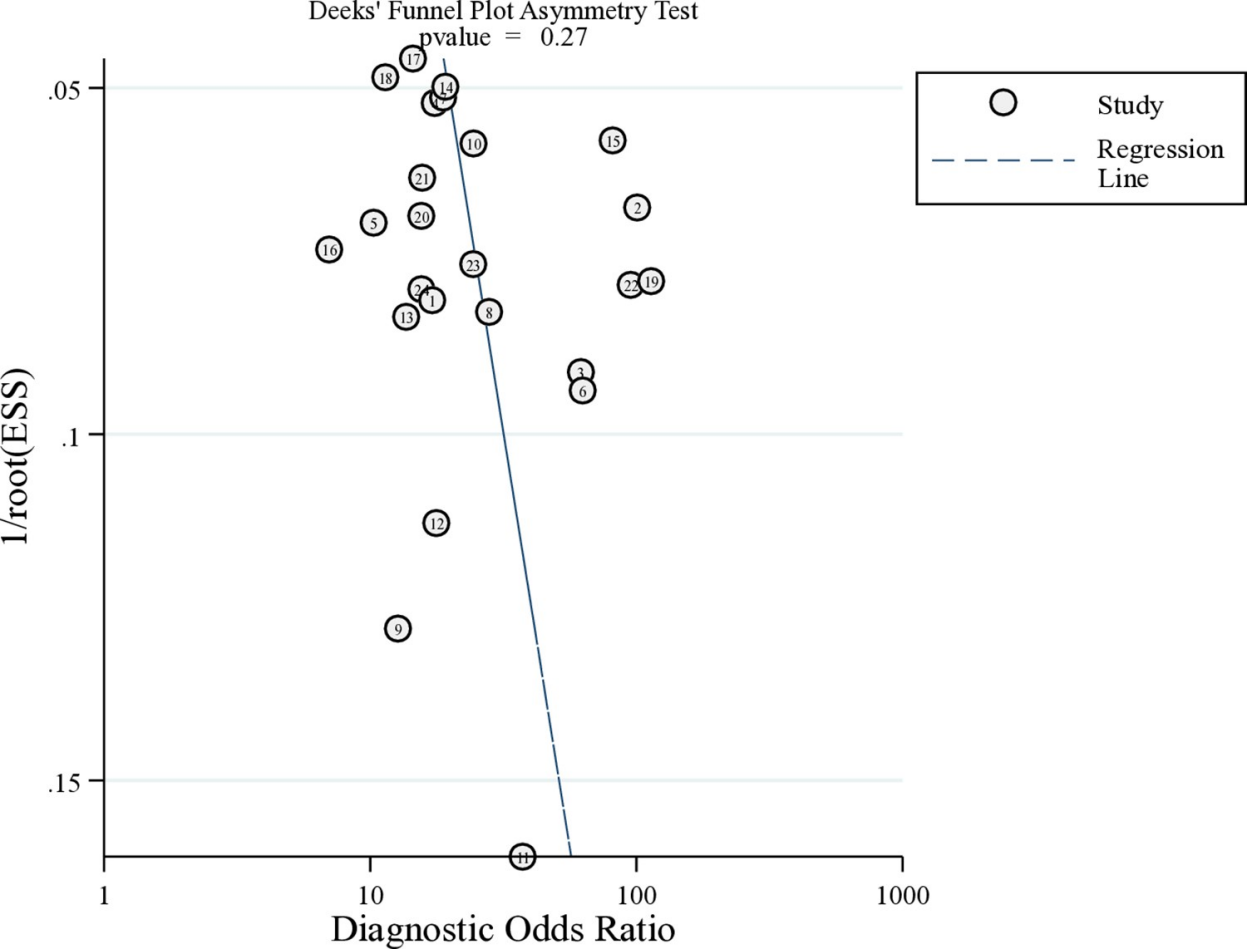
Deek’s funnel plot asymmetry test for publication bias.

A sensitivity analysis was conducted to assess the influence of each study on overall results (S4 Table). The pooled estimates for sensitivity, specificity, PLR, NLR, DOR, and AUC of the SROC curve remained stable after the exclusion of any single study, confirming the robustness of the findings. Publication bias was assessed using a funnel plot and asymmetry test, which showed no significant publication bias (P = 0.267, Fig 5).

### Clinical utility

**Fig 6:**
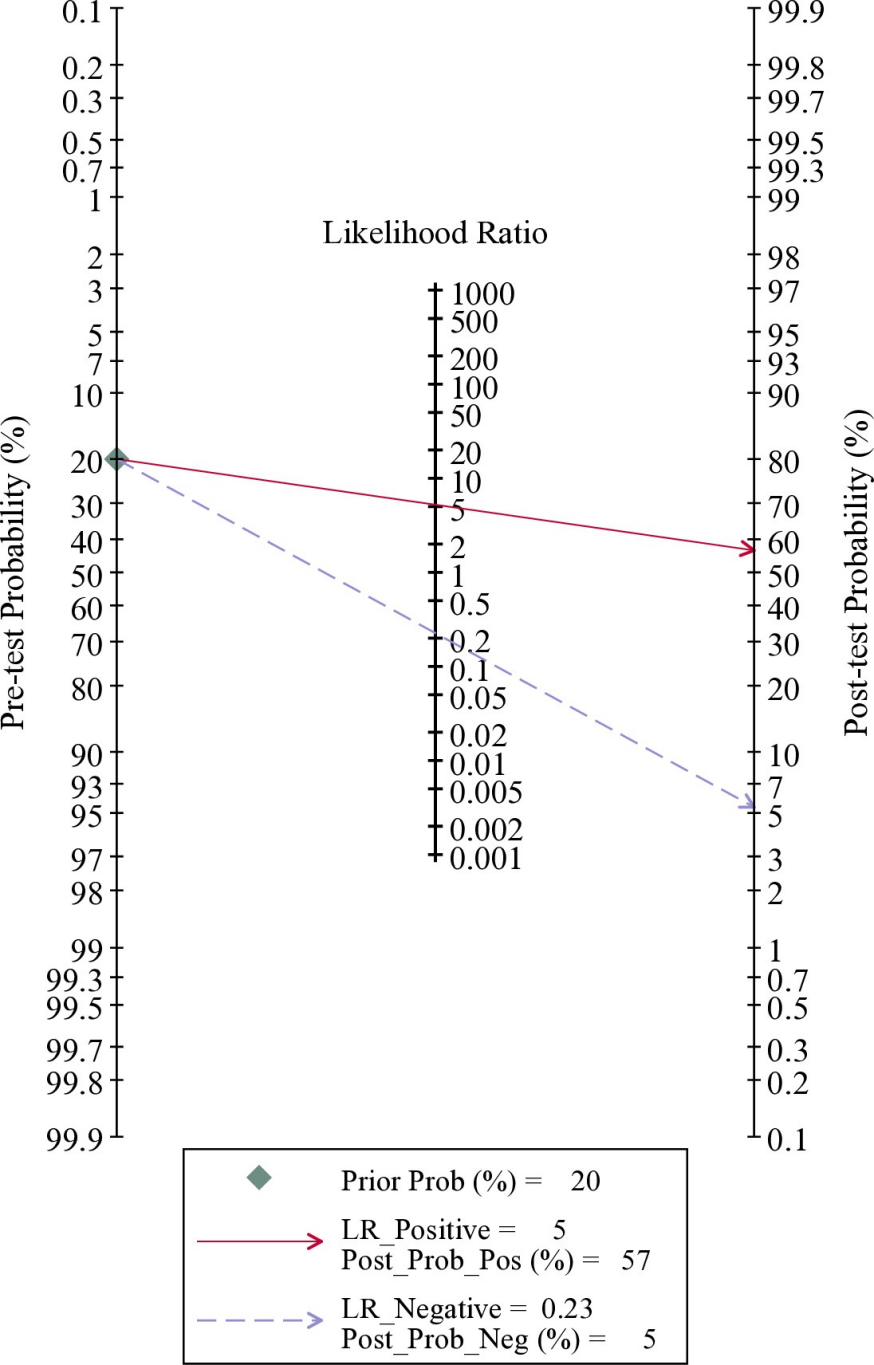
Fagan plot for assessing the clinical utility.

Using a DCE-MRI radiomics model, the posttest probability would rise from 20% to 57% with a PLR of 5 when the pretest result was positive (Fig 6). Conversely, the posttest probability would drop from 20% to 5% with an NLR of 0.23 when the pretest result was negative (Fig 6).

## Discussion

In this meta-analysis, we incorporated data from 24 studies encompassing 5588 breast cancer patients to assess the diagnostic performance of DCE-MRI radiomics in characterizing ALNM and SLNM. The prediction accuracy of DCE-MRI radiomics was notable, with pooled sensitivity, specificity, DOR, and overall AUC of 0.81 (95%CI: 0.77–0.84), 0.85 (95%CI: 0.81–0.87), 23.16 (95%CI: 17.20–31.19), and 0.90 (95%CI: 0.87–0.92), respectively. These findings indicate that DCE-MRI radiomics is an effective and accurate tool for predicting lymph node metastasis, holding significant clinical value for personalized treatment strategies in breast cancer patients.

However, substantial heterogeneity was noted in the sensitivity (I^2^ = 74.64%) and specificity (I^2^ = 83.18%) analyses. The threshold effect was ruled out as a source of heterogeneity, as indicated by the non-significant Spearman’s correlation coefficient (P = 0.538). Univariate meta-regression and subgroup analyses identified potential sources of heterogeneity. Notably, most studies were based on single-center data, with only three performing external validation or testing [10, 24, 35]. Diagnostic performance was lower in two-center studies compared to single-center studies, with reduced sensitivity and specificity. These findings indicated that the reproducibility of radiomic models developed by these single-center studies requires validation across multiple external datasets.

DCE-MRI radiomic models demonstrated lower pooled sensitivity and specificity for SLNM prediction compared to ALNM prediction. The future development of more effective tolls for SLNM prediction is essential. Most studies utilized SVM or LR algorithms to construct prediction models. SVM, which is robust to model misspecification and handles high-dimensional data effectively [40], showed better predictive ability than LR in subgroup analysis, with higher sensitivity and slightly higher specificity. Thus, SVM appears preferable over LR for radiomic model construction.

Peritumoral regions provide valuable insight into the tumor microenvironment relevant to tumor growth and invasion [41, 42]. Peritumoral radiomics have been effective in predicting breast lesion malignancy [43], molecular subtype [44], and response to neoadjuvant chemotherapy [45]. Most radiomic studies have focused on only intratumoral regions without considering peritumoral radiomic signatures. This meta-analysis included seven studies that combined intratumoral and peritumoral radiomic signatures for predicting tumor metastasis [16, 22, 26, 30, 34, 37, 38]. Among these studies, combined models demonstrated higher diagnostic yield than those using only intratumoral or peritumoral signatures [16, 30, 34, 37]. Subgroup analysis of our study showed that the combined radiomic models had higher sensitivity (0.84 *vs* 0.79) and only slightly lower specificity (0.83 *vs* 0.85) compared to intratumoral radiomic models. The pooled DOR for combined models was higher than that for intratumoral models (25.92 [95%CI: 13.44–50.00] *vs* 22.12 [95%CI: 16.03–30.53]), suggesting that adding peritumoral signatures enhances diagnostic performance of DCE-MRI radiomics.

Imaging at 3.0T offers a higher signal-to-noise ratio, resulting in better spatial resolution and image quality compared to 1.5T [46]. This meta-analysis found that 3.0T imaging had significantly higher sensitivity (0.83 *vs* 0.74) and slightly lower specificity (0.84 *vs* 0.86) than 1.5T. The DOR value was also higher for 3.0T imaging than that for 1.5T imaging (26.74 [95%CI: 18.11–39.49] *vs* 16.59 [95%CI: 12.46–22.09]), indicating superior discriminating ability of 3.0T imaging.

Our meta-analysis has several advantages over previous studies. Earlier meta-analyses focused on multiple MRI sequences, including T1-weighted image (T1WI), T2-weighted fat-suppressed (T2-FS), diffusion-weighted imaging (DWI), and DCE-MRI [47–50]. Diagnostic performance can vary significantly across different MRI sequences. For example, Chen C *et al*. found that the DCE sequence had higher pooled sensitivity and DOR than T2-FS and DWI [47]. A recent meta-analysis focused on DCE-MRI radiomics but included only 13 studies and 1618 participants [51]. Our study, focusing solely on DCE-MRI, provides more specific and valuable clinical guidance. Additionally, our study, with a 3.5-fold larger sample size, demonstrated better diagnostic yields and narrower confidence intervals compared to previous meta-analysis [51], leading to more reliable conclusions regarding the diagnostic performance of DCE-MRI radiomics in predicting ALNM/SLNM.

Nonetheless, this meta-analysis has limitations. All studies are retrospective, which introduce potential selection bias. Most studies were single-center and conducted in Chinese populations, lacking external validation in multiple centers and in other populations. Therefore, the reproducibility and generalizability of radiomic models need future investigations in prospective, multi-center studies across different populations. Variations in image processing aspects, such as ROIs, MRI protocols, DCE phases, tumor segmentation, feature selection, and model construction, contribute to substantial heterogeneity and reduce reproducibility. Standards and best practice need to be established. Additionally, we selected the best-performing model from each study, which may overestimate diagnostic performance.

## Conclusions

Our meta-analysis indicates that DCE-MRI radiomic models have good diagnostic performance in predicting ALNM and SLNM in breast cancer patients. Future prospective, large-scale, multi-center studies are needed to validate the effectiveness and clinical utility of this non-invasive method.

## Supporting information

S1 Checklist PRISMA-DTA checklist. (DOCX)

S1 Table List of articles identified by literature search. (DOCX)

S2 Table Analytical data extracted from included studies. (XLSX)

S3 Table Quality assessment using METRICS. (XLSX)

S4 Table Results of sensitivity analysis. (DOCX)
