# Supplementary material for: Diagnostic performance of DCE-MRI radiomics in predicting axillary lymph node metastasis in breast cancer patients: A meta-analysis
Source: PLoS One. 2024 Dec 3;19(12):e0314653. doi: 10.1371/journal.pone.0314653 (PMC11614294; doi:10.1371/journal.pone.0314653)
Supplement: S1 Table — (DOCX) [file pone.0314653.s002.docx]

S1 Table. List of articles identified by literature search.

| No. | Study | Inclusion/exclusion |
| --- | --- | --- |
| 1 | Arefan D, Chai RM, Sun M, Zuley ML, Wu SD. Machine learning prediction of axillary lymph node metastasis in breast cancer: 2D versus 3D radiomic features. MEDICAL PHYSICS. 2020;47(12):6334-42. | Included |
| 2 | Chen DX, Liu X, Hu CL, Hao RT, Wang OC, Xiao YL. Radiomics-based signature of breast cancer on preoperative contrast-enhanced MRI to predict axillary metastasis. FUTURE ONCOLOGY. 2022. | Included |
| 3 | Chen J, Zhu H, Gao J, Ge Y, Wang M, Li Y, et al. Radiomics models based on clinical-pathology and conventional and functional MRI for predicting lymph node metastases of breast cancer axillary. Chinese Journal of Medical Imaging Technology. 2021;37(6):885-90. | Included |
| 4 | Chen W, Lin G, Kong C, Wu X, Hu Y, Chen M, et al. Non-invasive prediction model of axillary lymph node status in patients with early-stage breast cancer: a feasibility study based on dynamic contrast-enhanced-MRI radiomics. The British journal of radiology. 2024;97(1154):439-50. | Included |
| 5 | Cheng Y, Xu S, Wang H, Wang X, Niu S, Luo Y, et al. Intra- and peri-tumoral radiomics for predicting the sentinel lymph node metastasis in breast cancer based on preoperative mammography and MRI. Frontiers in Oncology. 2022;12. | Included |
| 6 | Cui X, Wang N, Zhao Y, Chen S, Li S, Xu M, et al. Preoperative Prediction of Axillary Lymph Node Metastasis in Breast Cancer using Radiomics Features of DCE-MRI. Scientific reports. 2019;9(1):2240. | Included |
| 7 | Han L, Zhu Y, Liu Z, Yu T, He C, Jiang W, et al. Radiomic nomogram for prediction of axillary lymph node metastasis in breast cancer. European Radiology. 2019;29(7):3820-9. | Included |
| 8 | Liu C, Ding J, Spuhler K, Gao Y, Serrano Sosa M, Moriarty M, et al. Preoperative prediction of sentinel lymph node metastasis in breast cancer by radiomic signatures from dynamic contrast-enhanced MRI. Journal of Magnetic Resonance Imaging. 2019;49(1):131-40. | Included |
| 9 | Liu J, Sun D, Chen L, Fang Z, Song W, Guo D, et al. Radiomics analysis of dynamic contrast-enhanced magnetic resonance imaging for the prediction of sentinel lymph node metastasis in breast cancer. Frontiers in Oncology. 2019;9(SEP). | Included |
| 10 | Liu Y, Li X, Zhu L, Zhao Z, Wang T, Zhang X, et al. Preoperative Prediction of Axillary Lymph Node Metastasis in Breast Cancer Based on Intratumoral and Peritumoral DCE-MRI Radiomics Nomogram. Contrast Media and Molecular Imaging. 2022;2022. | Included |
| 11 | Ma M, Jiang Y, Qin N, Zhang X, Zhang Y, Wang X, et al. A Radiomics Model for Preoperative Predicting Sentinel Lymph Node Metastasis in Breast Cancer Based on Dynamic Contrast-Enhanced MRI. Frontiers in Oncology. 2022;12. | Included |
| 12 | Santucci D, Faiella E, Cordelli E, Sicilia R, de Felice C, Zobel BB, et al. 3T MRI-radiomic approach to predict for lymph node status in breast cancer patients. Cancers. 2021;13(9). | Included |
| 13 | Shan YN, Xu W, Wang R, Wang W, Pang PP, Shen QJ. A Nomogram Combined Radiomics and Kinetic Curve Pattern as Imaging Biomarker for Detecting Metastatic Axillary Lymph Node in Invasive Breast Cancer. Frontiers in Oncology. 2020;10. | Included |
| 14 | Song D, Yang F, Zhang Y, Guo Y, Qu Y, Zhang X, et al. Dynamic contrast-enhanced MRI radiomics nomogram for predicting axillary lymph node metastasis in breast cancer. Cancer Imaging. 2022;22(1). | Included |
| 15 | Tang Y, Chen L, Qiao Y, Li W, Deng R, Liang M. Radiomic Signature Based on Dynamic Contrast-Enhanced MRI for Evaluation of Axillary Lymph Node Metastasis in Breast Cancer. Computational and Mathematical Methods in Medicine. 2022;2022. | Included |
| 16 | Wang C, Chen X, Luo H, Liu Y, Meng R, Wang M, et al. Development and Internal Validation of a Preoperative Prediction Model for Sentinel Lymph Node Status in Breast Cancer: Combining Radiomics Signature and Clinical Factors. Frontiers in Oncology. 2021;11. | Included |
| 17 | Wang Q, Lin Y, Ding C, Guan W, Zhang X, Jia J, et al. Multi-modality radiomics model predicts axillary lymph node metastasis of breast cancer using MRI and mammography. European Radiology. 2024. | Included |
| 18 | Wang Y, Shang Y, Guo Y, Hai M, Gao Y, Wu Q, et al. Clinical study on the prediction of ALN metastasis based on intratumoral and peritumoral DCE-MRI radiomics and clinico-radiological characteristics in breast cancer. Front Oncol. 2024;14:1357145. | Included |
| 19 | Zhan C, Hu Y, Wang X, Liu H, Xia L, Ai T. Prediction of Axillary Lymph Node Metastasis in Breast Cancer using Intra-peritumoral Textural Transition Analysis based on Dynamic Contrast-enhanced Magnetic Resonance Imaging. Academic Radiology. 2022;29:S107-S15. | Included |
| 20 | Zhang J, Zhang Z, Mao N, Zhang H, Gao J, Wang B, et al. Radiomics nomogram for predicting axillary lymph node metastasis in breast cancer based on DCE-MRI: A multicenter study. Journal of X-ray science and technology. 2023;31(2):247-63. | Included |
| 21 | Zhu Y, Yang L, Shen H. Value of the Application of CE-MRI Radiomics and Machine Learning in Preoperative Prediction of Sentinel Lymph Node Metastasis in Breast Cancer. Frontiers in Oncology. 2021;11. | Included |
| 22 | 张成孟, 丁治民, 陈鹏, 刘奇峰. 基于DCE-MRI 瘤内及瘤周影像组学特征的机器学习模型预测乳腺癌腋窝淋巴结转移的价值. 中国医学计算机成像杂志. 2023;29:618-24. | Included |
| 23 | 赵楠楠, 朱芸, 汤晓敏, 李阳 张舒妮, 王玲玲, et al. 基于瘤内及瘤周MRI 影像组学列线图预测乳腺癌腋窝淋巴结转移. 磁共振成像. 2023;14(3):81-87. | Included |
| 24 | 朱永琪, 纪华, 朱彦芳, 吕静, 刘云. 术前MRI影像组学列线图对乳腺癌腋窝淋巴结转移的预测价值. 磁共振成像. 2022;13(5):52-8. | Included |
| 25 | Cattell R, Ying J, Lei L, Ding J, Chen SL, Sosa MS, et al. Preoperative prediction of lymph node metastasis using deep learning-based features. VISUAL COMPUTING FOR INDUSTRY BIOMEDICINE AND ART. 2022;5(1). | Excluded (duplicated samples) |
| 26 | Chai RM, Ma H, Xu MJ, Arefan D, Cui XY, Liu Y, et al. Differentiating axillary lymph node metastasis in invasive breast cancer patients: A comparison of radiomic signatures from multiparametric breast MR sequences. JOURNAL OF MAGNETIC RESONANCE IMAGING. 2019;50(4):1125-32. | Excluded (insufficient data) |
| 27 | Chen H, Lan X, Yu T, Li L, Tang S, Liu S, et al. Development and validation of a radiogenomics model to predict axillary lymph node metastasis in breast cancer integrating MRI with transcriptome data: A multicohort study. Frontiers in Oncology. 2022;12. | Excluded (data from public databases) |
| 28 | Chen H, Wang X, Lan X, Yu T, Li L, Tang S, et al. A radiomics model development via the associations with genomics features in predicting axillary lymph node metastasis of breast cancer: a study based on a public database and single-centre verification. Clinical Radiology. 2023;78(3):e279-e87. | Excluded (data from public databases) |
| 29 | Chen Y, Li J, Zhang J, Yu Z, Jiang H. Radiomic Nomogram for Predicting Axillary Lymph Node Metastasis in Patients with Breast Cancer. Academic Radiology. 2024;31(3):788-99. | Excluded (insufficient data) |
| 30 | Chen Y, Wang L, Dong X, Luo R, Ge Y, Liu H, et al. Deep Learning Radiomics of Preoperative Breast MRI for Prediction of Axillary Lymph Node Metastasis in Breast Cancer. Journal of digital imaging. 2023;36(4):1323-31. | Excluded (insufficient data) |
| 31 | Li L, Yu T, Sun J, Jiang S, Liu D, Wang X, et al. Prediction of the number of metastatic axillary lymph nodes in breast cancer by radiomic signature based on dynamic contrast-enhanced MRI. Acta Radiologica. 2022;63(8):1014-22. | Excluded (insufficient data) |
| 32 | Liu M, Mao N, Ma H, Dong J, Zhang K, Che K, et al. Pharmacokinetic parameters and radiomics model based on dynamic contrast enhanced MRI for the preoperative prediction of sentinel lymph node metastasis in breast cancer. Cancer Imaging. 2020;20(1). | Excluded (insufficient data) |
| 33 | Luo HB, Liu YY, Wang CH, Qing HM, Wang M, Zhang X, et al. Radiomic features of axillary lymph nodes based on pharmacokinetic modeling DCE-MRI allow preoperative diagnosis of their metastatic status in breast cancer. PLoS ONE. 2021;16(3 March). | Excluded (insufficient data) |
| 34 | Mao N, Dai Y, Lin F, Ma H, Duan S, Xie H, et al. Radiomics Nomogram of DCE-MRI for the Prediction of Axillary Lymph Node Metastasis in Breast Cancer. Frontiers in Oncology. 2020;10. | Excluded (insufficient data) |
| 35 | Wang D, Hu Y, Zhan C, Zhang Q, Wu Y, Ai T. A nomogram based on radiomics signature and deep-learning signature for preoperative prediction of axillary lymph node metastasis in breast cancer. Frontiers in Oncology. 2022;12. | Excluded (insufficient data) |
| 36 | Yu YF, Tan YJ, Xie CM, Hu QG, Ouyang J, Chen YJ, et al. Development and Validation of a Preoperative Magnetic Resonance Imaging Radiomics-Based Signature to Predict Axillary Lymph Node Metastasis and Disease-Free Survival in Patients With Early-Stage Breast Cancer. JAMA NETWORK OPEN. 2020;3(12). | Excluded (insufficient data) |
| 37 | Zhang X, Yang Z, Cui W, Zheng C, Li H, Li Y, et al. Preoperative prediction of axillary sentinel lymph node burden with multiparametric MRI-based radiomics nomogram in early-stage breast cancer. European Radiology. 2021;31(8):5924-39. | Excluded (ALN burden prediction) |
| 38 | 刘梅婕, 毛宁, 马恒, 史英红, 董建军, 杨平, et al. 基于影像组学构建乳腺癌前哨淋巴结转移预测模型的研究. 中国中西医结合影像学杂志. 2020;18(3):227-31 | Excluded (insufficient data) |
| 39 | 夏旭东,段成洲,李铭,等.基于MRI影像组学列线图预测乳腺癌腋窝淋巴结转移. 磁共振成像,2022,13(01):118-22 | Excluded (combined with other MRI sequences) |
| 40 | 朱娅娣,郁义星,杨玲.动态增强MRI影像组学模型术前预测乳腺癌前哨淋巴结转移.国际医学放射学杂志,2021,44(06):632-7. | Excluded (duplicated samples) |
| 41 | Abdel-Fatah TM, Ball G, Chen X, Mehaisi D, Giannotti E, Auer D, et al. Utilising artificial intelligence (AI) for analysing multiplex genomic and magnetic resonance imaging (MRI) data to develop multimodality predictive system for personalised neoadjuvant treatment of breast cancer (BC). Cancer Research. 2022;82(4 SUPPL). | Excluded (title and abstract review) |
| 42 | Allajbeu I, Hickman SE, Payne N, Moyle P, Taylor K, Sharma N, et al. Automated Breast Ultrasound: Technical Aspects, Impact on Breast Screening, and Future Perspectives. Current Breast Cancer Reports. 2021;13(3):141-50. | Excluded (title and abstract review) |
| 43 | Alongi P, Rovera G, Stracuzzi F, Popescu CE, Minutoli F, Arnone G, et al. Artificial Intelligence in Breast Cancer: A Systematic Review on PET Imaging Clinical Applications. Current Medical Imaging. 2023;19(8):832-43. | Excluded (title and abstract review) |
| 44 | Bitencourt AGV, Gibbs P, Saccarelli CR, Daimiel I, Lo Gullo R, Fox MJ, et al. MRI-based machine learning radiomics can predict HER2 expression level and pathologic response after neoadjuvant therapy in HER2 overexpressing breast cancer. EBIOMEDICINE. 2020;61. | Excluded (title and abstract review) |
| 45 | Boughdad S, Dirand AS, Orlhac F, Nioche C, Champion L, Buvat I. Prediction of complete pathological response after neoadjuvant chemotherapy in breast cancer using texture analysis: Comparison of FDG PETCT and DCE-MRI. Journal of Nuclear Medicine. 2018;59. | Excluded (title and abstract review) |
| 46 | Braman N. Radiomics For Surgical Planning and Prognostication. JAMA Netw Open. 2020;3(12):e2028608. | Excluded (title and abstract review) |
| 47 | Calabrese A, Santucci D, Landi R, Zobel BB, Faiella E, de Felice C. Radiomics MRI for lymph node status prediction in breast cancer patients: the state of art. JOURNAL OF CANCER RESEARCH AND CLINICAL ONCOLOGY. 2021;147(6):1587-97. | Excluded (title and abstract review) |
| 48 | Campana A, Gandomkar Z, Giannotti N, Reed W. The use of radiomics in magnetic resonance imaging for the pre-treatment characterisation of breast cancers: A scoping review. Journal of Medical Radiation Sciences. 2023;70(4):462-78. | Excluded (title and abstract review) |
| 49 | Chaudhury B, Zhou M, Goldgof DB, Hall LO, Gatenby RA, Gillies RJ, et al. Identifying Metastatic Breast Tumors Using Textural Kinetic Features of Contrast Based Habitat in DCE-MRI. MEDICAL IMAGING 2015: COMPUTER-AIDED DIAGNOSIS2015. | Excluded (title and abstract review) |
| 50 | Che S, Xue M, Li J, Tian Y, Hu J, Wang S, et al. Preoperative prediction of Ki‑67 expression status in breast cancer based on dynamic contrast enhanced MRI radiomics combined with clinical imaging features model. Chinese Journal of Radiology (China). 2022;56(9):967-75. | Excluded (title and abstract review) |
| 51 | Chen C, Qin YH, Chen HT, Zhu DY, Gao FB, Zhou XY. A meta-analysis of the diagnostic performance of machine learning-based MRI in the prediction of axillary lymph node metastasis in breast cancer patients. INSIGHTS INTO IMAGING. 2021;12(1). | Excluded (title and abstract review) |
| 52 | Chen M, Kong C, Lin G, Chen W, Guo X, Chen Y, et al. Development and validation of convolutional neural network-based model to predict the risk of sentinel or non-sentinel lymph node metastasis in patients with breast cancer: a machine learning study. eClinicalMedicine. 2023;63. | Excluded (title and abstract review) |
| 53 | Chen Y, Jiang J, Shi J, Chang W, Shi J, Chen M, et al. Dual-mode ultrasound radiomics and intrinsic imaging phenotypes for diagnosis of lymph node lesions. Annals of Translational Medicine. 2020;8(12). | Excluded (title and abstract review) |
| 54 | Chen Y, Liu X, Wang R, Lin X, Zhong J, Chen Y, et al. Ultrasound Radiomics in Breast Cancer - A Literature Review. Advanced Ultrasound in Diagnosis and Therapy. 2021;5(1):12-7. | Excluded (title and abstract review) |
| 55 | Cho P, Park CS, Park GE, Kim SH, Kim HS, Oh SJ. Diagnostic Usefulness of Diffusion-Weighted MRI for Axillary Lymph Node Evaluation in Patients with Breast Cancer. DIAGNOSTICS. 2023;13(3). | Excluded (title and abstract review) |
| 56 | Conti M, Morciano F, Bufi E, D’Angelo A, Panico C, Di Paola V, et al. Surgical Planning after Neoadjuvant Treatment in Breast Cancer: A Multimodality Imaging-Based Approach Focused on MRI. Cancers. 2023;15(5). | Excluded (title and abstract review) |
| 57 | Crivelli P, Ledda RE, Parascandolo N, Fara A, Soro D, Conti M. A New Challenge for Radiologists: Radiomics in Breast Cancer. BioMed Research International. 2018;2018. | Excluded (title and abstract review) |
| 58 | Dasgupta A, Bhardwaj D, DiCenzo D, Fatima K, Osapoetra LO, Quiaoit K, et al. Radiomics in predicting recurrence for patients with locally advanced breast cancer using quantitative ultrasound. Oncotarget. 2021;12(25):2437-48. | Excluded (title and abstract review) |
| 59 | Deike-Hofmann K, Kuder T, König F, Paech D, Dreher C, Delorme S, et al. Diffusion-weighted breast imaging. RADIOLOGE. 2018;58:14-9. | Excluded (title and abstract review) |
| 60 | Demircioglu A, Grueneisen J, Ingenwerth M, Hoffmann O, Pinker-Domenig K, Morris E, et al. A rapid volume of interest-based approach of radiomics analysis of breast MRI for tumor decoding and phenotyping of breast cancer. PLoS ONE. 2020;15(6 June). | Excluded (title and abstract review) |
| 61 | Di Paola V, Mazzotta G, Pignatelli V, Bufi E, D'Angelo A, Conti M, et al. Beyond N Staging in Breast Cancer: Importance of MRI and Ultrasound-based Imaging. CANCERS. 2022;14(17). | Excluded (title and abstract review) |
| 62 | Dietzel M, Trimboli RM, Zanardo M, Schultz-Wendtland R, Uder M, Clauser P, et al. The potential of predictive and prognostic breast MRI (P2-bMRI). European Radiology Experimental. 2022;6(1). | Excluded (title and abstract review) |
| 63 | Ding J, Chen S, Serrano Sosa M, Cattell R, Lei L, Sun J, et al. Optimizing the Peritumoral Region Size in Radiomics Analysis for Sentinel Lymph Node Status Prediction in Breast Cancer. Academic Radiology. 2022;29:S223-S8. | Excluded (title and abstract review) |
| 64 | Dong Y, Feng Q, Yang W, Lu Z, Deng C, Zhang L, et al. Preoperative prediction of sentinel lymph node metastasis in breast cancer based on radiomics of T2-weighted fat-suppression and diffusion-weighted MRI. European Radiology. 2018;28(2):582-91. | Excluded (title and abstract review) |
| 65 | Drukker K, Doyle C, Edwards A, Papaioannou J, Giger M. Radiomics for breast MRI in pre-treatment prediction of nodal response to neoadjuvant chemotherapy in node-positive breast cancer patients; a pilot study. Medical Physics. 2018;45(6):e183. | Excluded (title and abstract review) |
| 66 | Drukker K, Edwards A, Doyle C, Papaioannou J, Kulkarni K, Giger M. Pre-treatment prediction by hormone receptor subtype of response to neoadjuvant chemotherapy in node-positive breast cancer patients; a radiomics study. Medical Physics. 2019;46(6):e173-e4. | Excluded (title and abstract review) |
| 67 | Drukker K, Edwards A, Doyle C, Papaioannou J, Kulkarni K, Giger ML. Breast MRI radiomics for the pretreatment prediction of response to neoadjuvant chemotherapy in node-positive breast cancer patients. Journal of Medical Imaging. 2019;6(3). | Excluded (title and abstract review) |
| 68 | Eldaly AS, Avila FR, Torres-Guzman RA, Maita K, Garcia JP, Serrano LP, et al. Radiomics and Artificial Intelligence in Predicting Axillary Lymph Node Metastasis in Breast Cancer: A Systematic Review. Current Medical Imaging. 2023;19(6):564-78. | Excluded (title and abstract review) |
| 69 | Elshafeey N, Hwang KP, Adrada BE, Candelaria RP, Boge M, Mahmoud RM, et al. Radiomics model based on magnetic resonance image compilation (MagIC) as early predictor of pathologic complete response to neoadjuvant systemic therapy in triple-negative breast cancer. Cancer Research. 2022;82(4 SUPPL). | Excluded (title and abstract review) |
| 70 | Fang C, Zhang J, Li J, Shang H, Li K, Jiao T, et al. Clinical-radiomics nomogram for identifying HER2 status in patients with breast cancer: A multicenter study. Frontiers in Oncology. 2022;12. | Excluded (title and abstract review) |
| 71 | Feng B, Liu Z, Liu Y, Chen Y, Zhou H, Cui E, et al. Predicting lymphovascular invasion in clinically node-negative breast cancer detected by abbreviated magnetic resonance imaging: Transfer learning vs. radiomics. Frontiers in Oncology. 2022;12. | Excluded (title and abstract review) |
| 72 | Feng W, Gao Y, Lu XR, Xu YS, Guo ZZ, Lei JQ. Correlation between molecular prognostic factors and magnetic resonance imaging intravoxel incoherent motion histogram parameters in breast cancer. Magnetic Resonance Imaging. 2022;85:262-70. | Excluded (title and abstract review) |
| 73 | Fozza A, De Rose F, De Santis MC, Meattini I, Meduri B, D’angelo E, et al. Technological advancements and future perspectives in breast cancer radiation therapy. Expert Review of Anticancer Therapy. 2023;23(4):407-19. | Excluded (title and abstract review) |
| 74 | Gan L, Ma M, Liu Y, Liu Q, Xin L, Cheng Y, et al. A Clinical–Radiomics Model for Predicting Axillary Pathologic Complete Response in Breast Cancer With Axillary Lymph Node Metastases. Frontiers in Oncology. 2021;11. | Excluded (title and abstract review) |
| 75 | Gao J, Zhong X, Li W, Li Q, Shao H, Wang Z, et al. Attention-based Deep Learning for the Preoperative Differentiation of Axillary Lymph Node Metastasis in Breast Cancer on DCE-MRI. Journal of Magnetic Resonance Imaging. 2023;57(6):1842-53. | Excluded (title and abstract review) |
| 76 | Gong X, Guo Y, Zhu T, Peng X, Xing D, Zhang M. Diagnostic performance of radiomics in predicting axillary lymph node metastasis in breast cancer: A systematic review and meta-analysis. Frontiers in Oncology. 2022;12. | Excluded (title and abstract review) |
| 77 | Gravina M, Cordelli E, Santucci D, Soda P, Sansone C, Ieee. Evaluating Tumour Bounding Options for Deep Learning-based Axillary Lymph Node Metastasis Prediction in Breast Cancer. 2022 26TH INTERNATIONAL CONFERENCE ON PATTERN RECOGNITION (ICPR)2022. p. 4335-42. | Excluded (title and abstract review) |
| 78 | Groheux D, Ulaner GA, Hindie E. Breast cancer: treatment response assessment with FDG-PET/CT in the neoadjuvant and in the metastatic setting. Clinical and Translational Imaging. 2023;11(5):439-52. | Excluded (title and abstract review) |
| 79 | Gu J, Jiang T. Ultrasound radiomics in personalized breast management: Current status and future prospects. Frontiers in Oncology. 2022;12. | Excluded (title and abstract review) |
| 80 | Gu JH, Tong T, Xu D, Cheng F, Fang CY, He C, et al. Deep learning radiomics of ultrasonography for comprehensively predicting tumor and axillary lymph node status after neoadjuvant chemotherapy in breast cancer patients: A multicenter study. CANCER. 2023;129(3):356-66. | Excluded (title and abstract review) |
| 81 | Guo L, Du S, Gao S, Zhao R, Huang G, Jin F, et al. Delta-Radiomics Based on Dynamic Contrast-Enhanced MRI Predicts Pathologic Complete Response in Breast Cancer Patients Treated with Neoadjuvant Chemotherapy. Cancers. 2022;14(14). | Excluded (title and abstract review) |
| 82 | Guo LC, Du SY, Yang XP, Li S, Zhang LN. The role of magnetic resonance imaging on evaluating response of neoadjuvant therapy for breast cancer. Chinese Journal of Academic Radiology. 2020;3(3):125-36. | Excluded (title and abstract review) |
| 83 | Guo X, Liu Z, Sun C, Zhang L, Wang Y, Li Z, et al. Deep learning radiomics of ultrasonography: Identifying the risk of axillary non-sentinel lymph node involvement in primary breast cancer. EBioMedicine. 2020;60. | Excluded (title and abstract review) |
| 84 | Guo Y, Zhang H, Yuan LL, Chen WD, Zhao HB, Yu QQ, et al. Machine learning and new insights for breast cancer diagnosis. JOURNAL OF INTERNATIONAL MEDICAL RESEARCH. 2024;52(4). | Excluded (title and abstract review) |
| 85 | Han X, Cao W, Wu L, Liang C. Radiomics Assessment of the Tumor Immune Microenvironment to Predict Outcomes in Breast Cancer. Frontiers in Immunology. 2021;12. | Excluded (title and abstract review) |
| 86 | Haraguchi T, Kobayashi Y, Hirahara D, Kobayashi T, Takaya E, Nagai MT, et al. Radiomics model of diffusion-weighted whole-body imaging with background signal suppression (DWIBS) for predicting axillary lymph node status in breast cancer. Journal of X-ray science and technology. 2023;31(3):627-40. | Excluded (title and abstract review) |
| 87 | Heacock L, Lewin A, Ayoola A, Moccaldi M, Babb JS, Kim SG, et al. Dynamic Contrast-Enhanced MRI Evaluation of Pathologic Complete Response in Human Epidermal Growth Factor Receptor 2 (HER2)-Positive Breast Cancer After HER2-Targeted Therapy. Academic Radiology. 2020;27(5):e87-e93. | Excluded (title and abstract review) |
| 88 | Hou Y, Jiang KW, Wang LL, Zhi R, Bao ML, Li Q, et al. Biopsy-free AI-aided precision MRI assessment in prediction of prostate cancer biochemical recurrence. BRITISH JOURNAL OF CANCER. 2023;129(10):1625-33. | Excluded (title and abstract review) |
| 89 | Huang Y, Zhu T, Zhang X, Li W, Zheng X, Cheng M, et al. Longitudinal MRI-based fusion novel model predicts pathological complete response in breast cancer treated with neoadjuvant chemotherapy: a multicenter, retrospective study. eClinicalMedicine. 2023;58. | Excluded (title and abstract review) |
| 90 | Imbriaco M, Ponsiglione A. Predicting pathologic complete response after neoadjuvant chemotherapy. Radiology. 2021;299(2):301-2. | Excluded (title and abstract review) |
| 91 | Ji J, Ju S, Cai W. Editorial: Radiomics-based theranostics in cancer precision medicine. Frontiers in Oncology. 2023;13. | Excluded (title and abstract review) |
| 92 | Jiang T, Song J, Wang X, Niu S, Zhao N, Dong Y, et al. Intratumoral and Peritumoral Analysis of Mammography, Tomosynthesis, and Multiparametric MRI for Predicting Ki-67 Level in Breast Cancer: a Radiomics-Based Study. Molecular Imaging and Biology. 2022;24(4):550-9. | Excluded (title and abstract review) |
| 93 | Jiang W, Meng R, Cheng Y, Wang H, Han T, Qu N, et al. Intra- and Peritumoral Based Radiomics for Assessment of Lymphovascular Invasion in Invasive Breast Cancer. Journal of Magnetic Resonance Imaging. 2024;59(2):613-25. | Excluded (title and abstract review) |
| 94 | Kahán Z. Diversity of breast cancers begins at imaging…. European Journal of Radiology. 2022;154. | Excluded (title and abstract review) |
| 95 | Kayadibi Y, Kocak B, Ucar N, Akan YN, Yildirim E, Bektas S. MRI Radiomics of Breast Cancer: Machine Learning-Based Prediction of Lymphovascular Invasion Status. Acad Radiol. 2022;29 Suppl 1:S126-s34. | Excluded (title and abstract review) |
| 96 | Kim EE. Preface. Current Medical Imaging. 2022;18(1):1. | Excluded (title and abstract review) |
| 97 | Kong X, Zhang Q, Wu X, Zou T, Duan J, Song S, et al. Advances in Imaging in Evaluating the Efficacy of Neoadjuvant Chemotherapy for Breast Cancer. Frontiers in Oncology. 2022;12. | Excluded (title and abstract review) |
| 98 | Krome S. Predict lymphovascular invasion with Radiomics. RoFo Fortschritte auf dem Gebiet der Rontgenstrahlen und der Bildgebenden Verfahren. 2020;192(7):626-7. | Excluded (title and abstract review) |
| 99 | la Forgia D, Armenio A, Cutrignelli D, de Santis V, Fanizzi A, Maiorella A, et al. MRI role in the periprosthetic lymphoma screening: a case report. International Journal of Radiation Research. 2021;19(4):1055-8. | Excluded (title and abstract review) |
| 100 | Lee HJ, Nguyen AT, Song MW, Lee JE, Park SB, Jeong WG, et al. Prediction of Residual Axillary Nodal Metastasis Following Neoadjuvant Chemotherapy for Breast Cancer: Radiomics Analysis Based on Chest Computed Tomography. Korean Journal of Radiology. 2023;24(6):498-511. | Excluded (title and abstract review) |
| 101 | Lee HJ, Park JH, Nguyen AT, Do LN, Park MH, Lee JS, et al. Prediction of the histologic upgrade of ductal carcinoma in situ using a combined radiomics and machine learning approach based on breast dynamic contrast-enhanced magnetic resonance imaging. Frontiers in Oncology. 2022;12. | Excluded (title and abstract review) |
| 102 | Lee SH, Park H, Ko ES. Radiomics in breast imaging from techniques to clinical applications: A review. Korean Journal of Radiology. 2020;21(7):779-92. | Excluded (title and abstract review) |
| 103 | Li B, Zhu T, Liu Z, Wang K, Tian J. Multi-omics fusion for prediction of response to neoadjuvant therapy in breast cancer with external validation. Cancer Research. 2022;82(4 SUPPL). | Excluded (title and abstract review) |
| 104 | Li C, Li W, Liu CY, Zheng HR, Cai J, Wang SS. Artificial intelligence in multiparametric magnetic resonance imaging: A review. MEDICAL PHYSICS. 2022;49(10):E1024-E54. | Excluded (title and abstract review) |
| 105 | Li CL, Song LR, Yin JD. Intratumoral and Peritumoral Radiomics Based on Functional Parametric Maps from Breast DCE-MRI for Prediction of HER-2 and Ki-67 Status. JOURNAL OF MAGNETIC RESONANCE IMAGING. 2021;54(3):703-14. | Excluded (title and abstract review) |
| 106 | Li N, Song C, Cui G, Huang X, Zhang H, Su J, et al. Optimized Radiomics Nomogram Based on Automated Breast Ultrasound System: A Potential Tool for Preoperative Prediction of Metastatic Lymph Node Burden in Breast Cancer. Breast Cancer: Targets and Therapy. 2023;15:121-32. | Excluded (title and abstract review) |
| 107 | Li Q, Huang Y, Xiao Q, Duan S, Wang S, Li J, et al. Value of radiomics based on CE-MRI for predicting the efficacy of neoadjuvant chemotherapy in invasive breast cancer. British Journal of Radiology. 2022;95(1139). | Excluded (title and abstract review) |
| 108 | Li X, Yang L, Jiao X. Comparison of Traditional Radiomics, Deep Learning Radiomics and Fusion Methods for Axillary Lymph Node Metastasis Prediction in Breast Cancer. Academic Radiology. 2023;30(7):1281-7. | Excluded (title and abstract review) |
| 109 | Li Y, Yang ZL, Lv WZ, Qin YJ, Tang CL, Yan X, et al. Non-Mass Enhancements on DCE-MRI: Development and Validation of a Radiomics-Based Signature for Breast Cancer Diagnoses. Frontiers in Oncology. 2021;11. | Excluded (title and abstract review) |
| 110 | Li YL, Wang LZ, Shi QL, He YJ, Li JF, Zhu HT, et al. CT Radiomics for Predicting Pathological Complete Response of Axillary Lymph Nodes in Breast Cancer After Neoadjuvant Chemotherapy: A Prospective Study. ONCOLOGIST. 2023. | Excluded (title and abstract review) |
| 111 | Lin G, Chen W, Fan Y, Zhou Y, Li X, Hu X, et al. Machine Learning Radiomics-Based Prediction of Non-sentinel Lymph Node Metastasis in Chinese Breast Cancer Patients with 1-2 Positive Sentinel Lymph Nodes: A Multicenter Study. Academic Radiology. 2024. | Excluded (title and abstract review) |
| 112 | Lin G, Wang X, Ye H, Cao W. Radiomic Models Predict Tumor Microenvironment Using Artificial Intelligence—the Novel Biomarkers in Breast Cancer Immune Microenvironment. Technology in Cancer Research and Treatment. 2023;22. | Excluded (title and abstract review) |
| 113 | Liqing YU, Huang Z, Xiao Z, Tang X, Zeng Z, Tang X, et al. Unveiling the best predictive models for early‑onset metastatic cancer: Insights and innovations (Review). Oncology Reports. 2024;51(4). | Excluded (title and abstract review) |
| 114 | Liu CJ, Zhang L, Sun Y, Geng L, Wang R, Shi KM, et al. Application of CT and MRI images based on an artificial intelligence algorithm for predicting lymph node metastasis in breast cancer patients: a meta-analysis. BMC CANCER. 2023;23(1). | Excluded (title and abstract review) |
| 115 | Liu JJ, Wang XC, Hu MS, Zheng Y, Zhu L, Wang W, et al. Development of an ultrasound-based radiomics nomogram to preoperatively predict Ki-67 expression level in patients with breast cancer. FRONTIERS IN ONCOLOGY. 2022;12. | Excluded (title and abstract review) |
| 116 | Liu Q, Hu P. Radiogenomic association of deep MR imaging features with genomic profiles and clinical characteristics in breast cancer. Biomarker Research. 2023;11(1). | Excluded (title and abstract review) |
| 117 | Liu S, Du S, Gao S, Teng Y, Jin F, Zhang L. A delta-radiomic lymph node model using dynamic contrast enhanced MRI for the early prediction of axillary response after neoadjuvant chemotherapy in breast cancer patients. BMC Cancer. 2023;23(1). | Excluded (title and abstract review) |
| 118 | Liu W, Chen W, Xia J, Lu Z, Fu Y, Li Y, et al. Lymph node metastasis prediction and biological pathway associations underlying DCE-MRI deep learning radiomics in invasive breast cancer. BMC Medical Imaging. 2024;24(1). | Excluded (title and abstract review) |
| 119 | Liu Z, Feng B, Li C, Chen Y, Chen Q, Li X, et al. Preoperative prediction of lymphovascular invasion in invasive breast cancer with dynamic contrast-enhanced-MRI-based radiomics. Journal of Magnetic Resonance Imaging. 2019;50(3):847-57. | Excluded (title and abstract review) |
| 120 | Liu ZY, Wang S, Dong D, Wei JW, Fang C, Zhou XZ, et al. The Applications of Radiomics in Precision Diagnosis and Treatment of Oncology: Opportunities and Challenges. THERANOSTICS. 2019;9(5):1303-22. | Excluded (title and abstract review) |
| 121 | Maforo N, Li H, Lan L, Edwards A, Giger ML. Prognostic radiomics of breast cancer on DCE and DWI MR images. Medical Physics. 2016;43(6 PART2):3378. | Excluded (title and abstract review) |
| 122 | Mainta IC, Sfakianaki I, Shiri I, Botsikas D, Garibotto V. The Clinical Added Value of Breast Cancer Imaging Using Hybrid PET/MR Imaging. Magnetic Resonance Imaging Clinics of North America. 2023;31(4):565-77. | Excluded (title and abstract review) |
| 123 | Manu MR, Poongodi T. PREDICTION OF THE TUMOR RESPONSE LYMPH NODE BASED ON DEEP RESIDUAL BOLTZMANN CONVOLUTION NEURAL NETWORK. NeuroQuantology. 2022;20(8):6788-800. | Excluded (title and abstract review) |
| 124 | Mao N, Yin P, Li Q, Wang QL, Liu MJ, Ma H, et al. Radiomics nomogram of contrast-enhanced spectral mammography for prediction of axillary lymph node metastasis in breast cancer: a multicenter study. EUROPEAN RADIOLOGY. 2020;30(12):6732-9. | Excluded (title and abstract review) |
| 125 | Mao N, Yin P, Zhang HC, Zhang K, Song XC, Xing D, et al. Mammography-based radiomics for predicting the risk of breast cancer recurrence: a multicenter study. BRITISH JOURNAL OF RADIOLOGY. 2021;94(1127). | Excluded (title and abstract review) |
| 126 | Marino MA, Avendano D, Zapata P, Riedl CC, Pinker K. Lymph Node Imaging in Patients with Primary Breast Cancer: Concurrent Diagnostic Tools. Oncologist. 2020;25(2):e231-e42. | Excluded (title and abstract review) |
| 127 | Mazurowski MA, Saha A, Harowicz MR, Cain EH, Marks JR, Marcom PK. Association of distant recurrence-free survival with algorithmically extracted MRI characteristics in breast cancer. J Magn Reson Imaging. 2019;49(7):e231-e40. | Excluded (title and abstract review) |
| 128 | Mendez AM, Fang LK, Meriwether CH, Batasin SJ, Loubrie S, Rodríguez-Soto AE, et al. Diffusion Breast MRI: Current Standard and Emerging Techniques. Frontiers in Oncology. 2022;12. | Excluded (title and abstract review) |
| 129 | Ming Y, Wu N, Qian T, Li X, Wan DQ, Li C, et al. Progress and Future Trends in PET/CT and PET/MRI Molecular Imaging Approaches for Breast Cancer. Frontiers in Oncology. 2020;10. | Excluded (title and abstract review) |
| 130 | Montemezzi S, Benetti G, Bisighin MV, Camera L, Zerbato C, Caumo F, et al. 3T DCE-MRI Radiomics Improves Predictive Models of Complete Response to Neoadjuvant Chemotherapy in Breast Cancer. Frontiers in Oncology. 2021;11. | Excluded (title and abstract review) |
| 131 | Moran CJ. Editorial for "Evaluating Tumor-Infiltrating Lymphocytes in Breast Cancer Using Preoperative MRI-based Radiomics". J Magn Reson Imaging. 2022;55(3):785-6. | Excluded (title and abstract review) |
| 132 | Morrell GR. Editorial for "Assessment of Lymphovascular Invasion in Breast Cancer Using a Combined MRI Morphological Features, Radiomics, and Deep Learning Approach Based on Dynamic Contrast-Enhanced MRI". J Magn Reson Imaging. 2024;59(6):2250-1. | Excluded (title and abstract review) |
| 133 | Obeid JP, Stoyanova R, Kwon D, Patel M, Padgett K, Slingerland J, et al. Multiparametric evaluation of preoperative MRI in early stage breast cancer: prognostic impact of peri-tumoral fat. Clinical and Translational Oncology. 2017;19(2):211-8. | Excluded (title and abstract review) |
| 134 | Oliveira C, Oliveira F, Vaz SC, Marques HP, Cardoso F. Prediction of pathological response after neoadjuvant chemotherapy using baseline FDG PET heterogeneity features in breast cancer. British Journal of Radiology. 2023;96(1146). | Excluded (title and abstract review) |
| 135 | Perelli F, Mattei A, Scambia G, Cavaliere AF. Editorial: Methods in gynecological oncology. Frontiers in Oncology. 2023;13. | Excluded (title and abstract review) |
| 136 | Pesapane F, De Marco P, Rapino A, Lombardo E, Nicosia L, Tantrige P, et al. How Radiomics Can Improve Breast Cancer Diagnosis and Treatment. JOURNAL OF CLINICAL MEDICINE. 2023;12(4). | Excluded (title and abstract review) |
| 137 | Pesapane F, Rotili A, Agazzi GM, Botta F, Raimondi S, Penco S, et al. Recent radiomics advancements in breast cancer: Lessons and pitfalls for the next future. Current Oncology. 2021;28(4):2351-72. | Excluded (title and abstract review) |
| 138 | Pierre K, Gupta M, Raviprasad A, Sadat Razavi SM, Patel A, Peters K, et al. Medical imaging and multimodal artificial intelligence models for streamlining and enhancing cancer care: opportunities and challenges. Expert Review of Anticancer Therapy. 2023;23(12):1265-79. | Excluded (title and abstract review) |
| 139 | Pujara AC, Kim E, Axelrod D, Melsaether AN. PET/MRI in Breast Cancer. Journal of Magnetic Resonance Imaging. 2019;49(2):328-42. | Excluded (title and abstract review) |
| 140 | Qi X, Wang W, Pan S, Liu G, Xia L, Duan S, et al. Predictive value of triple negative breast cancer based on DCE-MRI multi-phase full-volume ROI clinical radiomics model. Acta Radiologica. 2024;65(2):173-84. | Excluded (title and abstract review) |
| 141 | Qiu X, Fu Y, Ye Y, Wang Z, Cao C. A Nomogram Based on Molecular Biomarkers and Radiomics to Predict Lymph Node Metastasis in Breast Cancer. Frontiers in Oncology. 2022;12. | Excluded (title and abstract review) |
| 142 | Qiu Y, Zhang X, Wu Z, Wu S, Yang Z, Wang D, et al. MRI-Based Radiomics Nomogram: Prediction of Axillary Non-Sentinel Lymph Node Metastasis in Patients With Sentinel Lymph Node-Positive Breast Cancer. Frontiers in Oncology. 2022;12. | Excluded (title and abstract review) |
| 143 | Ravichandran K, Braman N, Janowczyk A, Madabhushi A. A deep learning classifier for prediction of pathological complete response to neoadjuvant chemotherapy from baseline breast DCE-MRI. MEDICAL IMAGING 2018: COMPUTER-AIDED DIAGNOSIS2018. | Excluded (title and abstract review) |
| 144 | Reig B, Ha R. Editorial on “Diagnosis of Benign and Malignant Breast Lesions on DCE-MRI by Using Radiomics and Deep Learning With Consideration of Peritumor Tissue”. Journal of Magnetic Resonance Imaging. 2020;51(3):810-1. | Excluded (title and abstract review) |
| 145 | Reig B, Heacock L, Geras KJ, Moy L. Machine learning in breast MRI. Journal of Magnetic Resonance Imaging. 2020;52(4):998-1018. | Excluded (title and abstract review) |
| 146 | Reig B, Heacock L, Lewin A, Cho N, Moy L. Role of MRI to Assess Response to Neoadjuvant Therapy for Breast Cancer. JOURNAL OF MAGNETIC RESONANCE IMAGING. 2020;52(6):1587-606. | Excluded (title and abstract review) |
| 147 | Reig B, Lewin AA, Du L, Heacock L, Toth HK, Heller SL, et al. Breast MRI for Evaluation of Response to Neoadjuvant Therapy. Radiographics : a review publication of the Radiological Society of North America, Inc. 2021;41(3):665-79. | Excluded (title and abstract review) |
| 148 | Rinneburger M, Carolus H, Iuga AI, Weisthoff M, Lennartz S, Hokamp NGß, et al. Automated localization and segmentation of cervical lymph nodes on contrast-enhanced CT using a 3D foveal fully convolutional neural network. European Radiology Experimental. 2023;7(1). | Excluded (title and abstract review) |
| 149 | Romeo V, Accardo G, Perillo T, Basso L, Garbino N, Nicolai E, et al. Assessment and prediction of response to neoadjuvant chemotherapy in breast cancer: A comparison of imaging modalities and future perspectives. Cancers. 2021;13(14). | Excluded (title and abstract review) |
| 150 | Romeo V, Kapetas P, Clauser P, Rasul S, Cuocolo R, Caruso M, et al. Simultaneous 18F-FDG PET/MRI Radiomics and Machine Learning Analysis of the Primary Breast Tumor for the Preoperative Prediction of Axillary Lymph Node Status in Breast Cancer. Cancers. 2023;15(20). | Excluded (title and abstract review) |
| 151 | Romeo V, Moy L, Pinker K. AI-Enhanced PET and MR Imaging for Patients with Breast Cancer. PET Clinics. 2023;18(4):567-75. | Excluded (title and abstract review) |
| 152 | Rubio IT, Sobrido C. Neoadjuvant approach in patients with early breast cancer: patient assessment, staging, and planning. Breast. 2022;62:S17-S24. | Excluded (title and abstract review) |
| 153 | Samiei S, Granzier RWY, Ibrahim A, Primakov S, Lobbes MBI, Beets-Tan RGH, et al. Dedicated axillary mri-based radiomics analysis for the prediction of axillary lymph node metastasis in breast cancer. Cancers. 2021;13(4):1-15. | Excluded (title and abstract review) |
| 154 | Sang L, Liu Z, Huang C, Xu J, Wang H. Multiparametric MRI-based radiomics nomogram for predicting the hormone receptor status of HER2-positive breast cancer. Clinical Radiology. 2024;79(1):60-6. | Excluded (title and abstract review) |
| 155 | Santucci D, Faiella E, Cordelli E, Calabrese A, Landi R, de Felice C, et al. The Impact of Tumor Edema on T2-Weighted 3T-MRI Invasive Breast Cancer Histological Characterization: A Pilot Radiomics Study. Cancers (Basel). 2021;13(18). | Excluded (title and abstract review) |
| 156 | Santucci D, Faiella E, Gravina M, Cordelli E, de Felice C, Zobel BB, et al. CNN-Based Approaches with Different Tumor Bounding Options for Lymph Node Status Prediction in Breast DCE-MRI. CANCERS. 2022;14(19). | Excluded (title and abstract review) |
| 157 | Satake H, Ishigaki S, Ito R, Naganawa S. Radiomics in breast MRI: current progress toward clinical application in the era of artificial intelligence. RADIOLOGIA MEDICA. 2022;127(1):39-56. | Excluded (title and abstract review) |
| 158 | Shaikh S. Editorial for “Attention-based Deep Learning for the Preoperative Differentiation of Axillary Lymph Node Metastasis in Breast Cancer on DCE-MRI”. Journal of Magnetic Resonance Imaging. 2023;57(6):1854-5. | Excluded (title and abstract review) |
| 159 | Shan Y, Gong X, Ding Z, Shen Q, Xu W, Pang P, et al. Dynamic contrast-enhanced MRI radiomic features predict axillary lymph node metastasis of breast cancer. Chinese Journal of Radiology (China). 2019;53(9):742-7. | Excluded (title and abstract review) |
| 160 | Sheng W, Xia S, Wang Y, Yan L, Ke S, Mellisa E, et al. Invasive ductal breast cancer molecular subtype prediction by MRI radiomic and clinical features based on machine learning. Frontiers in Oncology. 2022;12. | Excluded (title and abstract review) |
| 161 | Song B, Yang K, Garneau J, Lu C, Li L, Lee J, et al. Radiomic Features Associated With HPV Status on Pretreatment Computed Tomography in Oropharyngeal Squamous Cell Carcinoma Inform Clinical Prognosis. Frontiers in Oncology. 2021;11. | Excluded (title and abstract review) |
| 162 | Song SE, Woo OH, Cho Y, Cho KR, Park KH, Kim JW. Prediction of Axillary Lymph Node Metastasis in Early-stage Triple-Negative Breast Cancer Using Multiparametric and Radiomic Features of Breast MRI. Academic Radiology. 2023;30:S25-S37. | Excluded (title and abstract review) |
| 163 | Spuhler KD, Ding J, Liu C, Sun J, Serrano-Sosa M, Moriarty M, et al. Task-based assessment of a convolutional neural network for segmenting breast lesions for radiomic analysis. Magnetic Resonance in Medicine. 2019;82(2):786-95. | Excluded (title and abstract review) |
| 164 | Sun QC, Lin XN, Zhao YS, Li L, Yan K, Liang D, et al. Deep Learning vs. Radiomics for Predicting Axillary Lymph Node Metastasis of Breast Cancer Using Ultrasound Images: Don't Forget the Peritumoral Region. FRONTIERS IN ONCOLOGY. 2020;10. | Excluded (title and abstract review) |
| 165 | Tan H, Gan F, Wu Y, Zhou J, Tian J, Lin Y, et al. Preoperative Prediction of Axillary Lymph Node Metastasis in Breast Carcinoma Using Radiomics Features Based on the Fat-Suppressed T2 Sequence. Academic Radiology. 2020;27(9):1217-25. | Excluded (title and abstract review) |
| 166 | Tan HN, Wu YP, Bao FC, Zhou J, Wan JZ, Tian J, et al. Mammography-based radiomics nomogram: a potential biomarker to predict axillary lymph node metastasis in breast cancer. BRITISH JOURNAL OF RADIOLOGY. 2020;93(1111). | Excluded (title and abstract review) |
| 167 | Tan W, Yang M, Yang H, Zhou F, Shen W. Predicting the response to neoadjuvant therapy for early-stage breast cancer: Tumor-, blood-, and imaging-related biomarkers. Cancer Management and Research. 2018;10:4333-47. | Excluded (title and abstract review) |
| 168 | Tan Y, Yu Y, Liu J, He Z, Chen Y, Ren W, et al. Machine learning intratumoral and axillary lymph node magnetic resonance imaging radiomics for predicting axillary lymph node metastasis in patients with early-stage invasive breast cancer (RBC-01 Study). ANNALS OF ONCOLOGY. 2020;31:S1244-S5. | Excluded (title and abstract review) |
| 169 | Tang WJ, Kong QC, Cheng ZX, Liang YS, Jin Z, Chen LX, et al. Performance of radiomics models for tumour-infiltrating lymphocyte (TIL) prediction in breast cancer: the role of the dynamic contrast-enhanced (DCE) MRI phase. European Radiology. 2022;32(2):864-75. | Excluded (title and abstract review) |
| 170 | Tang Y, Che XL, Wang WJ, Su S, Nie Y, Yang CM. Radiomics model based on features of axillary lymphatic nodes to predict axillary lymphatic node metastasis in breast cancer. MEDICAL PHYSICS. 2022;49(12):7555-66. | Excluded (title and abstract review) |
| 171 | Umutlu L, Kirchner J, Bruckmann NM, Morawitz J, Antoch G, Ingenwerth M, et al. Multiparametric integrated18f-fdg pet/mri-based radiomics for breast cancer phenotyping and tumor decoding. Cancers. 2021;13(12). | Excluded (title and abstract review) |
| 172 | Vaz SC, Oliveira C, Teixeira R, Arias-Bouda LMP, Cardoso MJ, De Geus-Oei LF. The current role of nuclear medicine in breast cancer. British Journal of Radiology. 2023;96(1149). | Excluded (title and abstract review) |
| 173 | Wang C, Chen X, Hongbing L, Liu Y, Meng R, Liu S, et al. A nomogram for preoperatively predicting sentinel lymph node status in breast cancer based on DCE-MRI radiomic features and clinical factors. Insights into Imaging. 2022;14:379. | Excluded (title and abstract review) |
| 174 | Wang H, Zhao M, Fan X, Yu T, Xu S. The value of MRI radiomics features for prediction of lymphovascular invasion in invasive breast cancer. Chinese Journal of Radiology (China). 2022;56(9):982-8. | Excluded (title and abstract review) |
| 175 | Wang J, Gao XN, Zhang SX, Zhang Y. Machine-learning methods based on the texture and non-texture features of MRI for the preoperative prediction of sentinel lymph node metastasis in breast cancer. TRANSLATIONAL CANCER RESEARCH. 2023;12(12):3471-85. | Excluded (title and abstract review) |
| 176 | Wang JJ, Zhang XH, Guo XH, Ying Y, Wang X, Luan ZH, et al. Prediction of Lymphovascular Space Invision in Endometrial Cancer based on Multi-parameter MRI Radiomics Model. Curr Med Imaging. 2024. | Excluded (title and abstract review) |
| 177 | Wang K, Zhu T, Huang Y, Li W. Longitudinal MRI-based radiomic model to complement sentinel lymph node biopsy assessment after neoadjuvant chemotherapy in initially clinically node-positive breast cancer: A multicentre, diagnostic study. Journal of Clinical Oncology. 2023;41(16):e13623. | Excluded (title and abstract review) |
| 178 | Wang X, Xie T, Luo J, Zhou Z, Yu X, Guo X. Radiomics predicts the prognosis of patients with locally advanced breast cancer by reflecting the heterogeneity of tumor cells and the tumor microenvironment. Breast Cancer Research. 2022;24(1). | Excluded (title and abstract review) |
| 179 | Wang Y, Zhang L, Qi L, Yi XP, Li MH, Zhou M, et al. Machine Learning: Applications and Advanced Progresses of Radiomics in Endocrine Neoplasms. JOURNAL OF ONCOLOGY. 2021;2021. | Excluded (title and abstract review) |
| 180 | Wang ZJ, Sun H, Li J, Chen J, Meng FC, Li H, et al. Preoperative Prediction of Axillary Lymph Node Metastasis in Breast Cancer Using CNN Based on Multiparametric MRI. JOURNAL OF MAGNETIC RESONANCE IMAGING. 2022;56(3):700-9. | Excluded (title and abstract review) |
| 181 | Wekking D, Porcu M, De Silva P, Saba L, Scartozzi M, Solinas C. Breast MRI: Clinical Indications, Recommendations, and Future Applications in Breast Cancer Diagnosis. Current Oncology Reports. 2023;25(4):257-67. | Excluded (title and abstract review) |
| 182 | Wu J, Mayer AT, Li R. Integrated imaging and molecular analysis to decipher tumor microenvironment in the era of immunotherapy. Seminars in Cancer Biology. 2022;84:310-28. | Excluded (title and abstract review) |
| 183 | Wu Z, Lin Q, Song H, Chen J, Wang G, Fu G, et al. Evaluation of Lymphatic Vessel Invasion Determined by D2-40 Using Preoperative MRI-Based Radiomics for Invasive Breast Cancer. Academic Radiology. 2023;30(11):2458-68. | Excluded (title and abstract review) |
| 184 | Wu ZJ, Lin Q, Wang HB, Wang GQ, Fu GM, Bian TT. An MRI-Based Radiomics Nomogram to Distinguish Ductal Carcinoma In Situ with Microinvasion From Ductal Carcinoma In Situ of Breast Cancer. ACADEMIC RADIOLOGY. 2023;30:S71-S81. | Excluded (title and abstract review) |
| 185 | Xia B, Wang H, Wang Z, Qian Z, Xiao Q, Liu Y, et al. A Combined Nomogram Model to Predict Disease-free Survival in Triple-Negative Breast Cancer Patients With Neoadjuvant Chemotherapy. Frontiers in Genetics. 2021;12. | Excluded (title and abstract review) |
| 186 | Xu A, Chu X, Zhang S, Zheng J, Shi D, Lv S, et al. Development and validation of a clinicoradiomic nomogram to assess the HER2 status of patients with invasive ductal carcinoma. BMC Cancer. 2022;22(1). | Excluded (title and abstract review) |
| 187 | Xu A, Chu X, Zhang S, Zheng J, Shi D, Lv S, et al. Prediction Breast Molecular Typing of Invasive Ductal Carcinoma Based on Dynamic Contrast Enhancement Magnetic Resonance Imaging Radiomics Characteristics: A Feasibility Study. Frontiers in Oncology. 2022;12. | Excluded (title and abstract review) |
| 188 | Xu H, Dai Y, Ma Y, Shuai G, Zhang Y. MR T1WI intratumoral and peritumoral radiomics combined with clinical features for predicting effect of neoadjuvant chemotherapy for breast cancer. Chinese Journal of Medical Imaging Technology. 2023;39(10):1520-5. | Excluded (title and abstract review) |
| 189 | Xu ML, Zeng SE, Li F, Cui XW, Liu GF. Preoperative prediction of lymphovascular invasion in patients with T1 breast invasive ductal carcinoma based on radiomics nomogram using grayscale ultrasound. FRONTIERS IN ONCOLOGY. 2022;12. | Excluded (title and abstract review) |
| 190 | Xu N, Zhou J, He X, Ye S, Miao H, Liu H, et al. Radiomics Model for Evaluating the Level of Tumor-Infiltrating Lymphocytes in Breast Cancer Based on Dynamic Contrast-Enhanced MRI. Clin Breast Cancer. 2021;21(5):440-9.e1. | Excluded (title and abstract review) |
| 191 | Yang C, Li J, Wang W. A Clinical-Radiomics Model of Preoperative Prediction IMN Status of Breast Cancer Patients. International Journal of Radiation Oncology Biology Physics. 2022;114(3):e41. | Excluded (title and abstract review) |
| 192 | Yang X, Fan X, Lin S, Zhou Y, Liu H, Wang X, et al. Assessment of Lymphovascular Invasion in Breast Cancer Using a Combined MRI Morphological Features, Radiomics, and Deep Learning Approach Based on Dynamic Contrast-Enhanced MRI. J Magn Reson Imaging. 2024;59(6):2238-49. | Excluded (title and abstract review) |
| 193 | Yao HR, Li CC, Yu YF, Xie CM, Ouyang J, Tan YJ, et al. Radiomics multi-parametric MRI of before and after neoadjuvant chemotherapy associated with axillary lymph node metastasis and prognostic in patients with breast cancer: A multicenter RBC-002 study. CANCER RESEARCH. 2020;80(4). | Excluded (title and abstract review) |
| 194 | Ye DM, Wang HT, Yu T. The application of radiomics in breast MRI: A review. Technology in Cancer Research and Treatment. 2020;19(1):1-16. | Excluded (title and abstract review) |
| 195 | Yin Z, Cui Y, Ren J, Yang X. Preoperative predictive value of a multiparametric MRI radiomics for axillary lymph node metastasis of breast cancer. Chinese Journal of Anatomy and Clinics. 2023;28(12):773-81. | Excluded (title and abstract review) |
| 196 | Yoshida K, Kawashima H, Kannon T, Tajima A, Ohno N, Terada K, et al. Prediction of pathological complete response to neoadjuvant chemotherapy in breast cancer using radiomics of pretreatment dynamic contrast-enhanced MRI. Magnetic Resonance Imaging. 2022;92:19-25. | Excluded (title and abstract review) |
| 197 | Yu Y, He Z, Ouyang J, Tan Y, Chen Y, Gu Y, et al. Magnetic resonance imaging radiomics predicts preoperative axillary lymph node metastasis to support surgical decisions and is associated with tumor microenvironment in invasive breast cancer: A machine learning, multicenter study. EBioMedicine. 2021;69. | Excluded (title and abstract review) |
| 198 | Yu Y, Tan Y, Hu Q, Ouyang J, Chen Y, Yang G, et al. Development and validation of a magnetic resonance imaging radiomics-based signature to predict axillary lymph node metastasis and disease-free survival in patients with breast cancer: A multicenter cohort study. Annals of Oncology. 2020;31:S309. | Excluded (title and abstract review) |
| 199 | Yu Y, Wang Z, Wang Q, Su X, Li Z, Wang R, et al. Radiomic model based on magnetic resonance imaging for predicting pathological complete response after neoadjuvant chemotherapy in breast cancer patients. Frontiers in Oncology. 2023;13. | Excluded (title and abstract review) |
| 200 | Yu YY, Zhang R, Dong RT, Hu QY, Yu T, Liu F, et al. Feasibility of an ADC-based radiomics model for predicting pelvic lymph node metastases in patients with stage IB-IIA cervical squamous cell carcinoma. BRITISH JOURNAL OF RADIOLOGY. 2019;92(1097). | Excluded (title and abstract review) |
| 201 | Yuan J, Mingming M, Yuanjia C, Yingpu C, Changxin L, Yaofeng Z, et al. Feasibility study of predicting axillary lymph node metastasis of breast cancer using radiomics analysis based on dynamic contrast‑enhanced MRI. Chinese Journal of Radiology (China). 2022;56(6):631-5. | Excluded (title and abstract review) |
| 202 | Zhang B, Yu Y, Mao Y, Wang H, Lv M, Su X, et al. Development of MRI-Based Deep Learning Signature for Prediction of Axillary Response After NAC in Breast Cancer. Academic Radiology. 2024;31(3):800-11. | Excluded (title and abstract review) |
| 203 | Zhang C, Qi L, Cai J, Wu H, Xu Y, Lin Y, et al. Clinicomics-guided distant metastasis prediction in breast cancer via artificial intelligence. BMC Cancer. 2023;23(1). | Excluded (title and abstract review) |
| 204 | Zhang H, Niu S, Chen H, Wang L, Wang X, Wu Y, et al. Radiomics signatures for predicting the Ki-67 level and HER-2 status based on bone metastasis from primary breast cancer. Frontiers in Cell and Developmental Biology. 2023;11. | Excluded (title and abstract review) |
| 205 | Zhang J, Li L, Zhe X, Tang M, Zhang X, Lei X, et al. The Diagnostic Performance of Machine Learning-Based Radiomics of DCE-MRI in Predicting Axillary Lymph Node Metastasis in Breast Cancer: A Meta-Analysis. Frontiers in Oncology. 2022;12. | Excluded (title and abstract review) |
| 206 | Zhang J, Wu J, Zhou XS, Shi F, Shen D. Recent advancements in artificial intelligence for breast cancer: Image augmentation, segmentation, diagnosis, and prognosis approaches. Seminars in Cancer Biology. 2023;96:11-25. | Excluded (title and abstract review) |
| 207 | Zhang L, Shen M, Zhang D, He X, Du Q, Liu N, et al. Radiomics Nomogram Based on Dual-Sequence MRI for Assessing Ki-67 Expression in Breast Cancer. Journal of Magnetic Resonance Imaging. 2023. | Excluded (title and abstract review) |
| 208 | Zhang TY, Tan T, Samperna R, Li Z, Gao Y, Wang X, et al. Radiomics and artificial intelligence in breast imaging: a survey. ARTIFICIAL INTELLIGENCE REVIEW. 2023;56(SUPPL 1):857-92. | Excluded (title and abstract review) |
| 209 | Zhang X, Zhong L, Zhang B, Zhang L, Du H, Lu L, et al. The effects of volume of interest delineation on MRI-based radiomics analysis: Evaluation with two disease groups. Cancer Imaging. 2019;19(1). | Excluded (title and abstract review) |
| 210 | Zheng H, Jian L, Li L, Liu W, Chen W. Delta-Radiomics Based on Dynamic Contrast-Enhanced MRI for Predicting Lymphovascular Invasion in Invasive Breast Cancer. Acad Radiol. 2024;31(5):1762-72. | Excluded (title and abstract review) |
| 211 | Zhong JY, Lu JJ, Zhang GC, Mao SQ, Chen HD, Yin Q, et al. An overview of meta-analyses on radiomics: more evidence is needed to support clinical translation. INSIGHTS INTO IMAGING. 2023;14(1). | Excluded (title and abstract review) |
| 212 | Zhou J, Liu Z, Tan H, Wu Y, Bai Y, Fu F, et al. Value of multi-parameter MRI radiomics features in the preoperative prediction of triple-negative and non-triple-negative breast cancer. Chinese Journal of Radiology (China). 2020;54(12):1179-84. | Excluded (title and abstract review) |
| 213 | Zhou WJ, Zhang YD, Kong WT, Zhang CX, Zhang B. Preoperative prediction of axillary lymph node metastasis in patients with breast cancer based on radiomics of gray-scale ultrasonography. GLAND SURGERY. 2021;10(6):1989-2001. | Excluded (title and abstract review) |
| 214 | Zhu T, Huang YH, Li W, Zhang YM, Lin YY, Cheng MY, et al. Multifactor artificial intelligence model assists axillary lymph node surgery in breast cancer after neoadjuvant chemotherapy: multicenter retrospective cohort study. International journal of surgery (London, England). 2023;109(11):3383-94. | Excluded (title and abstract review) |
| 215 | 安绍宇. 影像技术在乳腺癌内乳淋巴结转移检测方面的研究进展. 医学影像学杂志. 2010;20(10):1562-4. | Excluded (title and abstract review) |
| 216 | 白洪忠, 冯光, 李雯. 核磁共振检查在乳腺疾病诊断中的价值及技术要点分析. 影像技术. 2019;31(5). | Excluded (title and abstract review) |
| 217 | 白宛鹭, 邢华, 李海滨. 基于深度学习的乳腺癌MRI诊断及亚型识别研究进展. 中国实验诊断学. 2022;26(06):924-6. | Excluded (title and abstract review) |
| 218 | 别建华. 核磁共振成像对乳腺癌诊断的价值研究. 环球中医药. 2015(S2). | Excluded (title and abstract review) |
| 219 | 曹天寿. 仿淋巴结水凝胶捕获肿瘤细胞外囊泡增强抗肿瘤免疫反应及抑制肿瘤转移的研究 2023. | Excluded (title and abstract review) |
| 220 | 曹夕娜. 多模态MRI技术对三阴性乳腺癌（TNBC）与非三阴性乳腺癌（nTNBC）的诊断价值. 名医. 2022(07):21-3. | Excluded (title and abstract review) |
| 221 | 车丹丹, 赫铁军, 娄雪峰, 隋强. 超声弹性成像及核磁共振成像的联合在乳腺良恶性肿物的临床应用. 锦州医科大学学报. 2023;44(01):49-53. | Excluded (title and abstract review) |
| 222 | 陈朝一, 许波, 吴英, 吴凯文. 医学图像处理中的注意力机制研究综述. 计算机工程与应用. 2022;58(05):23-33. | Excluded (title and abstract review) |
| 223 | 陈红梅, 于湛, 黄文起. 不典型乳腺癌的CT和MRI成像特征分析. 西南国防医药. 2015;25(7). | Excluded (title and abstract review) |
| 224 | 陈欢. 超声联合钼靶对早期浸润性乳腺癌豁免前哨淋巴结活检的价值分析 2021. | Excluded (title and abstract review) |
| 225 | 陈继鑫, 孔恒. 多模态乳腺肿瘤图像特征提取与分级诊断的研究进展. 岭南现代临床外科. 2020;20(06):789-95. | Excluded (title and abstract review) |
| 226 | 陈珊珊, 张洁莉, 王婷婷, 臧爱民, 李玉苗, 杨华. 1例乳腺癌脉络膜转移的诊断及治疗. 山东医药. 2020;60(33). | Excluded (title and abstract review) |
| 227 | 陈文静, 马财, 徐蕊, 张文馨, 燕桂新. 磁共振不同序列与纹理特征相关性对乳腺结节的诊断价值. 中国医学装备. 2018;15(09):50-3. | Excluded (title and abstract review) |
| 228 | 陈鑫龙, 叶凯, 周文策. 人工智能在胰腺疾病新型诊疗模式中的应用及进展. 中国医学物理学杂志. 2022;39(08):1049-56. | Excluded (title and abstract review) |
| 229 | 程浩. 早期乳腺癌腋窝淋巴结术前影像学评价及腋窝淋巴结转移危险因素分析 2019. | Excluded (title and abstract review) |
| 230 | 崔娜, 董磊. 超声诊断技术在乳腺癌早期诊断中的应用及检出率分析. 影像研究与医学应用. 2022;6(22):117-9. | Excluded (title and abstract review) |
| 231 | 代佳佳, 肖何, 张琴, 李松霖, 陈川, 王阁. 局部晚期直肠癌新辅助化放疗病理学缓解的预测基因分析. 医学研究生学报. 2019;32(06):606-12. | Excluded (title and abstract review) |
| 232 | 邓伟雄, 汤勇才, 张年伟, 常颖智, 罗时敏, 谭卫民. 乳腺癌患者血清血管内皮生长因子水平与临床病理特征及新辅助化疗疗效的相关性. 实用医学杂志. 2015;31(18). | Excluded (title and abstract review) |
| 233 | 丁恩慈, 赵炎, 徐文贵, 沈婕. 18F-FDG PET/CT在肝细胞癌肝移植术前及预后评估中应用的研究进展. 中华器官移植杂志. 2019(08):508-9-10-11-12. | Excluded (title and abstract review) |
| 234 | 董迪, 巩立鑫, 王坤, 张崇, 田捷. 影像组学的临床应用. 中国科学基金. 2021;35(01):85-91. | Excluded (title and abstract review) |
| 235 | 杜岚, 杨敬春, 常莹. 标准乳腺MRI与腋窝超声鉴别乳腺癌有无转移及晚期腋窝淋巴结病变的差异. 中国医药导报. 2017;14(35). | Excluded (title and abstract review) |
| 236 | 杜森, 赵森, 周青, 孙亮亮, 吴海滨. 血清长链非编码RNA小核仁RNA宿主基因3检测联合核磁共振弥散加权成像在乳腺癌诊断中的应用. 陕西医学杂志. 2022;51(08):1021-4+37. | Excluded (title and abstract review) |
| 237 | 杜文侠, 米凌玉, 胡军利, 孟庆冰, 白文文, 高恒波. 乳腺癌淋巴结转移误诊为蜂窝织炎1例. 中国医药导报. 2021;18(24):179-82+96. | Excluded (title and abstract review) |
| 238 | 杜欣, 余建群, 周燚. 核磁共振弥散加权成像联合血清肿瘤标志物检测在乳腺癌诊断中的应用. 实用医院临床杂志. 2020;17(3). | Excluded (title and abstract review) |
| 239 | 付兰, 杨晓棠. 三阴性乳腺癌患者磁共振成像征象与临床病理特征的分析. 肿瘤研究与临床. 2013(11). | Excluded (title and abstract review) |
| 240 | 高思远, 冯章志, 杨明, 方拥军. 分析T1WI信号强度直方图对儿童急性淋巴细胞白血病的诊断价值. 南京医科大学学报(自然科学版). 2021;41(09):1383-7. | Excluded (title and abstract review) |
| 241 | 宫文晔, 郑一琼, 李席如. 核磁共振成像在非哺乳期乳腺炎诊疗中的研究进展. 解放军医学院学报. 2023;44(10):1167-71. | Excluded (title and abstract review) |
| 242 | 龚娜, 李辉安, 朱冰洁. 动态增强磁共振成像与扩散加权成像组学联合对微小乳腺癌病灶的诊断价值. 实用临床医药杂志. 2023;27(05):21-5+54. | Excluded (title and abstract review) |
| 243 | 龚子健, 曾柔, 龚良庚, 彭云, 叶印泉. 基于MRI影像组学及PSA结合机器学习对前列腺中央区良恶性结节的鉴别诊断. 暨南大学学报(自然科学与医学版). 2022;43(02):205-11. | Excluded (title and abstract review) |
| 244 | 郭少伟, 李庆霞, 邱刚, 房保栓, 尹岳松, 王东苗. 乳腺癌改良根治术后2种放疗技术的剂量学差异. 医学研究生学报. 2019;32(10):1055-8. | Excluded (title and abstract review) |
| 245 | 郭少伟. 乳腺癌改良根治术后两种放疗技术及内乳区非计划性受量的研究 2019. | Excluded (title and abstract review) |
| 246 | 郝春芳, 王淑玲, 佟仲生. 1例转移性隐匿性乳腺癌的临床诊治. 中国肿瘤临床. 2013;40(1). | Excluded (title and abstract review) |
| 247 | 何通翔, 文冬琳, 冉君, 李小明. RSNA2021骨骼肌肉影像学. 放射学实践. 2022;37(02):145-51. | Excluded (title and abstract review) |
| 248 | 胡慧娟. 乳腺MRI常规成像、动态增强成像、扩散加权成像对良恶性病变的诊断评价 2007. | Excluded (title and abstract review) |
| 249 | 胡瑛. PET/CT与磁共振诊断乳腺癌的结果比较. 中国CT和MRI杂志. 2017;15(12). | Excluded (title and abstract review) |
| 250 | 胡莹, 李培恒, 刘儒鹏, 陈阳阳. 3.0T MRI联合FFDM检查对三阴性乳腺癌诊断符合率的影响. 哈尔滨医药. 2022;42(4). | Excluded (title and abstract review) |
| 251 | 黄洁. 联合超声、MRI及钼靶评估Luminal A型Luminal B型乳腺癌的诊断价值研究 2023. | Excluded (title and abstract review) |
| 252 | 黄金叶子, 黎英姿, 周雯. 影像组学在乳腺癌诊断及预后的应用进展. 中国CT和MRI杂志. 2023;21(03):174-7. | Excluded (title and abstract review) |
| 253 | 黄日升, 陈冠峰, 潘灿玉, 连涛. 乳腺癌新辅助化疗后肝损害的MRI诊断分析. 现代医用影像学. 2022;31(10). | Excluded (title and abstract review) |
| 254 | 姜原, 秦乃姗. 多参数MRI在乳腺癌诊疗中的应用及进展. 中华临床医师杂志(电子版). 2020;14(11):872-6. | Excluded (title and abstract review) |
| 255 | 蒋一逍, 曹利平, 张国强. 胰腺癌早期筛查的研究进展. 浙江医学. 2024;46(05):553-60. | Excluded (title and abstract review) |
| 256 | 金昱, 潘凯枫, 张艺宝, 李文庆. 人工智能在肿瘤三级预防中的应用 机遇与挑战. 中国肿瘤临床. 2021;48(21):1082-7. | Excluded (title and abstract review) |
| 257 | 赖木雅, 刘子霖, 谭学渊, 农盛. MRI影像组学预测乳腺癌腋窝淋巴结转移. 现代医用影像学. 2024;33(02):252-5+70. | Excluded (title and abstract review) |
| 258 | 黎明英, 李瑞雄. 浸润性乳腺癌影像学检查诊断及应用价值研究进展. 世界复合医学. 2023;9(06):191-4. | Excluded (title and abstract review) |
| 259 | 李登平, 李菲, 寇福景. 彩超结合核磁共振对乳腺癌淋巴结转移的应用分析. 中华临床医师杂志·电子版. 2016;10(7). | Excluded (title and abstract review) |
| 260 | 李海军. 新型共载过氧化氢酶和PD-1抗体的多功能复合水凝胶体系联合放疗在三阴性乳腺癌中的治疗研究 2021. | Excluded (title and abstract review) |
| 261 | 李惠民. MRI和PET/CT对乳腺癌新辅助化疗效果评估的系统评价和Meta分析 2019. | Excluded (title and abstract review) |
| 262 | 李杰. 多功能纳米粒联合放疗和免疫治疗在三阴乳腺癌治疗中的研究 2021. | Excluded (title and abstract review) |
| 263 | 李捷, 张艳君, 王建东, 李席如. 以腋窝淋巴结转移为首发症状的隐性乳腺癌诊治分析. 军医进修学院学报. 2011;32(5). | Excluded (title and abstract review) |
| 264 | 李锦玉. 血浆IgM型自身抗体在早期乳腺癌检测中的价值评价 2023. | Excluded (title and abstract review) |
| 265 | 李军. 高血糖与前列腺癌临床特征及恶性程度的相关性研究 2019. | Excluded (title and abstract review) |
| 266 | 李丽, 郑玉荣, 张静, 马玉荣, 梁娟, 刘光耀. MRI参数与三阴性乳腺癌组织病理学分级及淋巴结转移的相关性研究. 中国中西医结合影像学杂志. 2020;18(4). | Excluded (title and abstract review) |
| 267 | 李茜哲. 近红外响应纳米酶通过光热消融和缺氧逆转增强STING依赖的先天抗肿瘤免疫反应 2023. | Excluded (title and abstract review) |
| 268 | 李清蓉, 沈文彬. 宫颈癌淋巴结转移MRI特征分析. 影像研究与医学应用. 2022;6(22):53-5. | Excluded (title and abstract review) |
| 269 | 李汝锐, 曾辉, 冯结映, 赖文佳, 陈卫国. 动态对比增强磁共振成像对术前空芯针穿刺活检诊断为乳腺导管原位癌术后病理升级为乳腺浸润性导管癌的评估价值. 分子影像学杂志. 2024;47(03):231-6. | Excluded (title and abstract review) |
| 270 | 李双双, 侯震, 刘娟, 任伟, 万遂人, 闫婧. 影像组学分析与建模工具综述. 中国医学物理学杂志. 2018;35(09):1043-9. | Excluded (title and abstract review) |
| 271 | 李玮超. 乳腺专用磁共振在乳腺癌筛查、综合治疗中的应用价值 2019. | Excluded (title and abstract review) |
| 272 | 李小娟, 刘相辰, 刘建国, 张苑, 范晓宁, 李桂娥, et al. 联合多种检查方法在提高乳腺肿块体检效果的作用. 国际医药卫生导报. 2018;24(12). | Excluded (title and abstract review) |
| 273 | 李小玲, 黄文荣, 李开成. 肿块型乳腺癌多模态MRI影像表现及其与CerbB-2、ER、PR相关性分析. 中国CT和MRI杂志. 2023;21(09):111-3. | Excluded (title and abstract review) |
| 274 | 李晓达. X线摄影、超声及核磁共振检查对乳腺良、恶性病变的诊断价值 2013. | Excluded (title and abstract review) |
| 275 | 李晓璐, 霍立群, 顾军. 正电子发射计算机断层扫描/磁共振成像系统在乳腺癌诊疗中的研究进展. 实用临床医药杂志. 2023;27(24):115-9. | Excluded (title and abstract review) |
| 276 | 李雄伟, 龙笑, 俞楠泽. 腋网综合征研究进展. 医学综述. 2017;23(4). | Excluded (title and abstract review) |
| 277 | 李萱, 张可丽, 曹柳, 于海胜, 方义湖. 病理尸检在高职高专病理实践教学中的应用. 数理医药学杂志. 2019;32(12):1895-6. | Excluded (title and abstract review) |
| 278 | 李琰, 杜森, 周青, 赵森. 动态增强MRI参数在乳腺癌早期诊断及预后评估中的应用. 中国CT和MRI杂志. 2023;21(05):79-81. | Excluded (title and abstract review) |
| 279 | 李燕锋. 肿瘤靶向miR155递送系统构建与体内外评价 2023. | Excluded (title and abstract review) |
| 280 | 李莹, 王安武. 乳腺磁共振动态增强扫描协同超声及乳腺X射线摄影在乳腺癌筛查及腋窝淋巴结转移中的价值. 中国现代医生. 2023;61(01):47-51. | Excluded (title and abstract review) |
| 281 | 梁鲜凤, 铺尚广萍. 超声联合诊断评估乳腺癌淋巴结转移价值研究. 健康必读. 2020(1):111-2. | Excluded (title and abstract review) |
| 282 | 林辅谊. Ki--67在乳腺癌新辅助化疗中的预测价值 2018. | Excluded (title and abstract review) |
| 283 | 林惠卿, 钟华成. 全数字化乳腺X线摄影联合MRI检查在诊断乳腺占位性病变中的临床应用价值. 中国医疗设备. 2021;36(03):99-101+23. | Excluded (title and abstract review) |
| 284 | 刘奋德. T1a-bN0M0期乳腺癌患者列线图预后模型的构建 2022. | Excluded (title and abstract review) |
| 285 | 刘海燕, 黄信, 曲少华, 蒋光愉, 王宁霞. 乳腺湿疹样癌(Paget's病)7例临床分析. 暨南大学学报·自然科学与医学版. 2017;38(2). | Excluded (title and abstract review) |
| 286 | 刘宏博. 乳腺癌腋淋巴结临床分期(cN)的影像学评估方法的分析比较 2022. | Excluded (title and abstract review) |
| 287 | 刘沁峰, 张颖, 潘伟, 于斌, 王涛, 张恩科, et al. 影像组学在乳腺癌诊疗中的应用研究进展. 临床医学研究与实践. 2020;5(34):1-4. | Excluded (title and abstract review) |
| 288 | 刘蓉, 马兴英, 许达, 张俊清, 胥卉苹. 彩超结合核磁共振对乳腺癌淋巴结转移的诊断价值. 西南国防医药. 2015;25(7). | Excluded (title and abstract review) |
| 289 | 刘绍华, 彭蓉蓉, 李丰章, 陈杰. 基于增强MRI影像组学在乳腺癌腋窝淋巴结转移预测模型的研究. 江西医药. 2021;56(10):1615-8+38. | Excluded (title and abstract review) |
| 290 | 刘苏婉. 构建多功能碳量子点用于乳腺癌的光热免疫联合治疗 2022. | Excluded (title and abstract review) |
| 291 | 陆兴练. 乳腺癌诊断技术的研究进展. 大医生. 2022;7(18):123-6. | Excluded (title and abstract review) |
| 292 | 罗彭婷. 基于特征工程和深度学习的乳腺癌前哨淋巴结转移预测方法研究 2019. | Excluded (title and abstract review) |
| 293 | 罗作明, editor 比较分析18F-FDG PET/CT与MRI在乳腺癌诊断中的临床价值2016. | Excluded (title and abstract review) |
| 294 | 吕芳滨, 林丽君, 谢森淼, 周佰林. 磁共振成像联合钼靶对乳腺恶性肿瘤的诊断效能分析. 中国妇幼保健. 2021;36(22):5341-4. | Excluded (title and abstract review) |
| 295 | 吕向. 应用MR功能成像技术进行乳腺癌新辅助化疗疗效评估的优势研究 2020. | Excluded (title and abstract review) |
| 296 | 马洪艳. 早期乳腺癌患者前哨淋巴结活检术替代腋窝淋巴结清扫术安全性研究 2017. | Excluded (title and abstract review) |
| 297 | 马纪. 乳腺癌腋窝淋巴结的MRI表现与前哨淋巴结转移的相关性研究 2013. | Excluded (title and abstract review) |
| 298 | 马楠. 病理性乳头溢液患者低剂量CT乳管造影多模态成像临床研究 2023. | Excluded (title and abstract review) |
| 299 | 马彦云. 基于磁共振多模型扩散加权成像的乳腺癌精准诊疗关键问题研究 2020. | Excluded (title and abstract review) |
| 300 | 马永刚, 张堃, 黄斌, 万涛, 赵志友. 动态对比增强和扩散加权成像的多参数MRI模型预测乳腺癌肿瘤血管新生的价值. 临床和实验医学杂志. 2022;21(04):443-6. | Excluded (title and abstract review) |
| 301 | 门禹龙. MRI在乳腺肿瘤良恶性鉴别诊断中应用价值探讨. 现代医用影像学. 2018;27(8). | Excluded (title and abstract review) |
| 302 | 苗环, 许文森, 孙宇. 浸润性乳腺癌MRI征象与HER-2及Ki-67表达的相关性. 现代肿瘤医学. 2020;28(22). | Excluded (title and abstract review) |
| 303 | 缪嘉, 陈心朋, 张大宏. 影像组学在膀胱肿瘤研究中的应用进展. 浙江临床医学. 2020;22(09):1374-6. | Excluded (title and abstract review) |
| 304 | 牛会丛. 鞘内注射新型靶向性锆基金属有机框架用于治疗脑膜癌病及其作用机制研究 2022. | Excluded (title and abstract review) |
| 305 | 欧国军, 张旭. 核磁共振结合彩超对乳腺癌淋巴结转移的诊断效果以及诊断准确率. 中国社区医师. 2018;34(24). | Excluded (title and abstract review) |
| 306 | 欧阳伟炜. 磁感应热疗及热化疗治疗大鼠乳腺癌的疗效及对免疫功能的影响 2010. | Excluded (title and abstract review) |
| 307 | 潘广, 马立岩, 杜志泉, 李浩. 核磁共振联合彩超对乳腺癌淋巴结转移的诊断效果及准确性评价. 影像研究与医学应用. 2019(13). | Excluded (title and abstract review) |
| 308 | 彭澍. 动态增强磁共振联合DWI对乳腺癌淋巴结转移的诊断价值时. 饮食保健. 2021(28):257. | Excluded (title and abstract review) |
| 309 | 桑蝶. Ki67与乳腺癌临床病理特征及新辅助化疗疗效的相关性 2015. | Excluded (title and abstract review) |
| 310 | 沈珊珊, 李洪祯, 余媛媛. 中国胰腺癌高危人群早期筛查和监测共识意见(2021,南京). 临床肝胆病杂志. 2022;38(05):1016-22. | Excluded (title and abstract review) |
| 311 | 施明良, 陈国明, 甘斌, 周晟, 梁宇, 谢铮. MRI动态增强定量参数在乳腺癌早期诊断的临床研究. 当代医学. 2019;25(33):147-8. | Excluded (title and abstract review) |
| 312 | 宋慧玲, 崔艳芬, 杨晓棠. 动态增强磁共振成像纹理分析对乳腺癌新辅助化疗效果的预测与评估. 肿瘤研究与临床. 2020;32(08):562-8. | Excluded (title and abstract review) |
| 313 | 宋吉成. 不同影像学检查方法对乳腺癌术前综合评估的临床应用价值. 影像研究与医学应用. 2021;5(5). | Excluded (title and abstract review) |
| 314 | 宋佳成, 蒋晓婷, 张爱宁, 张晶, 陈婷. 影像组学联合临床病理特征预测早期宫颈癌无病生存期的效能. 南京医科大学学报(自然科学版). 2024;44(01):52-9. | Excluded (title and abstract review) |
| 315 | 宋学镠. 基于超声、钼靶、核磁共振三种影像学特征分层与联合评估乳腺肿瘤的研究 2023. | Excluded (title and abstract review) |
| 316 | 宋振江. MRI在乳腺癌腋窝前哨淋巴结转移中的诊断价值. 影像技术. 2020;32(1). | Excluded (title and abstract review) |
| 317 | 苏佳娜, 陈忠, 陈泽文, 梁长松, 郭晓婷, 郭庆禄. 磁共振成像在乳腺癌腋窝前哨淋巴结转移中的诊断价值. 实用医技杂志. 2020;27(2). | Excluded (title and abstract review) |
| 318 | 苏亚英, 石子馨, 张苗, 焦光丽, 杨飞, 崔书君. 基于DCE-MRI影像组学定量预测进展期宫颈鳞癌同步放化疗反应的价值. 河北北方学院学报(自然科学版). 2023;39(02):11-7. | Excluded (title and abstract review) |
| 319 | 孙矗, 沈赞. 超顺磁性氧化铁颗粒增强核磁共振结合CT淋巴造影技术在乳腺癌前哨淋巴结定位及性质评估方面的研究进展. 癌症进展. 2015(1). | Excluded (title and abstract review) |
| 320 | 孙琳琳, 张旭. 乳腺癌磁共振征象与生物学行为、分子分型的关系分析. 生命科学仪器. 2020;18(05):35-40. | Excluded (title and abstract review) |
| 321 | 孙爽. 实体瘤脑膜转移癌的临床特征及预后分析 2019. | Excluded (title and abstract review) |
| 322 | 孙哲, 李艳翠. 常用影像学检查方法在乳腺癌诊断中的应用及进展. 中国现代医生. 2022;60(05):192-6. | Excluded (title and abstract review) |
| 323 | 孙梓筌, 刘连新. 人工智能在肝癌诊断及治疗中的进展及前景. 医学综述. 2020;26(22):4421-4. | Excluded (title and abstract review) |
| 324 | 汤建燕, 张亚杰, 李晓兵. 乳腺癌人表皮生长因子受体2表达对乳腺癌临床诊断与预后评估的意义. 遵义医科大学学报. 2020;43(01):85-90. | Excluded (title and abstract review) |
| 325 | 唐诗慧, 范维, 张政, 王萍, 邓巧玲, 许培培, et al. 肿瘤坏死因子-α在乳腺肿瘤中的表达及其与影像学特征的关系. 现代生物医学进展. 2017;17(23). | Excluded (title and abstract review) |
| 326 | 唐竹晓, 徐丽娜, 孙召龙, 陈雯, 李瑞南. 应用DCE-MRI联合DWI序列评估乳腺癌术前新辅助化疗疗效的可行性研究. 中国CT和MRI杂志. 2021;19(7). | Excluded (title and abstract review) |
| 327 | 童文伟, 吴海燕, 刘玉军, 王红梅, 张艳君. 乳腺癌改良根治术后乳糜漏并感染一例. 中华乳腺病杂志（电子版）. 2014;8(3):26,33. | Excluded (title and abstract review) |
| 328 | 涂景恋. 三阴乳腺癌的磁共振与病理对照研究 2011. | Excluded (title and abstract review) |
| 329 | 汪永凤, 欧小红, 陈思元, 韦智晓. 影像组学分类甲状腺结节良恶性的研究进展. 中国医疗设备. 2023;38(07):170-6. | Excluded (title and abstract review) |
| 330 | 王保茎, 秦全波, 毛怡盛, 孔玲, 丁丹卉. 动态增强磁共振联合DWI对乳腺癌淋巴结转移的诊断价值. 实用癌症杂志. 2020;35(2). | Excluded (title and abstract review) |
| 331 | 王芳. 乳腺癌患者核磁共振成像征象与细胞生物学因子指标的相关性. 中国卫生工程学. 2021;20(6). | Excluded (title and abstract review) |
| 332 | 王红, editor 超声、X线和MR对三阴性乳腺癌影像特征及临床和病理研究2012. | Excluded (title and abstract review) |
| 333 | 王慧, 王成锋, 王靖, 高纪东, 吴铁城, 方仪, et al. 影像学检查手段在早期乳腺癌及其淋巴结评价方面的意义. 癌症进展. 2014(6). | Excluded (title and abstract review) |
| 334 | 王晋峰, 郭美琴, 孙万里, 张春明, 薛宁. 乳腺癌磁共振成像误诊和漏诊病例的临床、影像和病理学特点. 肿瘤研究与临床. 2013(11). | Excluded (title and abstract review) |
| 335 | 王京. 乳腺癌可视化模型的建立及双特异性重组腺病毒与紫杉醇协同抑制作用的研究 2020. | Excluded (title and abstract review) |
| 336 | 王君松, 赵海玲. 不同影像学方法对乳腺肿瘤诊断的对比分析. 中国妇幼健康研究. 2019;30(05):637-41. | Excluded (title and abstract review) |
| 337 | 王丽君, editor 误诊为乳腺癌的非哺乳期乳腺炎的MRI、X线及超声表现2012. | Excluded (title and abstract review) |
| 338 | 王领梅. ROC曲线评估血清CA125联合彩色多普勒高频超声检测早期乳腺癌的诊断效能. 当代医学. 2022;28(16):134-6. | Excluded (title and abstract review) |
| 339 | 王晴晴, 代志清, 刘高峰. T2WI联合ADC值诊断前列腺癌的价值及与危险程度、PSA的关系. 分子诊断与治疗杂志. 2022;14(02):304-8. | Excluded (title and abstract review) |
| 340 | 王盛申, 贾海龙. 核磁共振联合彩超对乳腺癌淋巴结转移的诊断效果及准确性评价研究. 特别健康. 2020(12):139. | Excluded (title and abstract review) |
| 341 | 王硕, 李骥, 彭飞. 磁共振联合乳腺癌分子分型预测新辅助化疗乳腺癌患者的预后. 中国煤炭工业医学杂志. 2023;26(01):32-7. | Excluded (title and abstract review) |
| 342 | 王硕. 多参数MRI影像组学模型与乳腺癌腋窝淋巴结转移相关性研究 2022. | Excluded (title and abstract review) |
| 343 | 王新. 影像学检查手段在早期乳腺癌及其淋巴结评价方面的意义. 自我保健. 2020(18):46. | Excluded (title and abstract review) |
| 344 | 王歆光, 王东民, 张澜波, 段学宁, 刘荫华. 腋窝无痛性肿物. 中国实用外科杂志. 2008;28(6):505-7. | Excluded (title and abstract review) |
| 345 | 王雪岩, 刘艳. 应用MRI扫描ADC值评估乳腺浸润性导管癌组织学分级及预后的可行性研究. 中国CT和MRI杂志. 2020;18(08):56-9. | Excluded (title and abstract review) |
| 346 | 王燕彬, 刘有云, 畅旭东. WB-DWI对于乳腺癌患者术后临床评价. 影像技术. 2024;36(1). | Excluded (title and abstract review) |
| 347 | 王一菲. T7介导的双敏感PAMAM/DOX递药系统联合免疫原性死亡的抗肿瘤研究 2021. | Excluded (title and abstract review) |
| 348 | 王颖, 王斌杰, 周依. 多模态MR成像在乳腺良恶性病变的诊断价值及其与乳腺癌预后的相关性分析. 河南大学学报(医学版). 2023;42(02):128-34. | Excluded (title and abstract review) |
| 349 | 王宇, 康骅. 精准治疗理念下乳腺癌患者腋窝淋巴结的评估与处理. 肿瘤防治研究. 2022;49(10). | Excluded (title and abstract review) |
| 350 | 王振跃. 3.0T磁共振弥散加权成像测定ADC值与前列腺癌患者组织学分级、预后的关系分析. 中国医疗器械信息. 2022;28(20):10-2. | Excluded (title and abstract review) |
| 351 | 吴斌. 隐匿性乳腺癌36例诊治分析. 中华肿瘤防治杂志. 2007;14(19). | Excluded (title and abstract review) |
| 352 | 吴国君. 腋窝淋巴结清扫术后上肢继发性淋巴水肿动物模型建立的实验研究 2017. | Excluded (title and abstract review) |
| 353 | 吴佳芮. MRI纹理分析在乳腺癌腋窝淋巴结转移预测中的应用. 医学信息. 2020;33(23):157-9. | Excluded (title and abstract review) |
| 354 | 吴佩琪, 刘于宝, 陈祉妍, 蔡海桃, 毛小明. 基于MRI的瘤周影像组学在肿瘤研究中的应用进展. 分子影像学杂志. 2023;46(01):164-9. | Excluded (title and abstract review) |
| 355 | 吴佩琪, 毛小明, 马捷, 刘于宝. CT瘤周影像组学在肿瘤诊疗中的研究进展. 中国CT和MRI杂志. 2023;21(11):175-8. | Excluded (title and abstract review) |
| 356 | 吴佩琪, 杨雅俪, 周妍璐, 郭粉玲, 毛小明. 乳腺良恶性病变鉴别的MRI影像组学预测模型构建：基于随机森林、支持向量机、逻辑回归分类器. 分子影像学杂志. 2021;44(05):764-70. | Excluded (title and abstract review) |
| 357 | 吴向东, 田林, 郝晓鹏. 肿块强化与非肿块强化乳腺癌患者的临床和病理特征对比分析. 中国临床新医学. 2022;15(11). | Excluded (title and abstract review) |
| 358 | 吴怡雯, 周晓华, 陈菲, 陈嘉瑶, 欧阳良艳, 陈诗雁, et al. 基于自动乳腺全容积扫查影像组学对预测乳腺癌腋窝淋巴结负荷的价值. 中国超声医学杂志. 2023;39(05):499-502. | Excluded (title and abstract review) |
| 359 | 相泓冰, 曹丽, 曾庆, 门永忠. 超声联合磁共振成像对乳腺肿块良恶性的鉴别诊断价值. 中国实用医刊. 2021;48(11):65-8. | Excluded (title and abstract review) |
| 360 | 肖栋, 梁明, 陈亮, 范志刚, 张亦磊, 翟超, et al. 激素受体及Her2状态对乳腺癌术前B超及MRI评估腋窝淋巴结状态准确性的影响分析. 中国现代普通外科进展. 2014;17(12):946-9. | Excluded (title and abstract review) |
| 361 | 谢四梅, 张安秦, 朱彩霞, 连臻强, 张嫣, 王颀. 触诊及影像学检查对乳腺癌腋淋巴结转移状况预测价值探讨. 中华肿瘤防治杂志. 2014;21(15). | Excluded (title and abstract review) |
| 362 | 熊健, 郑历明, 余汉城, 骆施. DCE-MRI定量参数与食管癌病理分级的相关性研究. 中国CT和MRI杂志. 2023;21(02):110-2. | Excluded (title and abstract review) |
| 363 | 熊倩倩, 王坤. 影像基因组学预测乳腺癌新辅助化疗疗效. 循证医学. 2017;17(03):184-9+92. | Excluded (title and abstract review) |
| 364 | 徐莉萍, 羽洪文, 谢筱晞, 邓海璇, 张裕超. 3.0T磁共振功能成像诊断乳腺癌的价值研究. 中国医学创新. 2014;11(11). | Excluded (title and abstract review) |
| 365 | 徐亮, 陈源锋, 贾岩龙, 林佳特, 丘金铭, 吴仁华. 基于MRI的类淋巴系统研究进展. 中国CT和MRI杂志. 2021;19(02):7-11. | Excluded (title and abstract review) |
| 366 | 徐树明, 程林仙, 杨宣琴, 原韶玲, 付兰. 囊实性乳腺癌的影像学及临床病理学特征. 肿瘤研究与临床. 2013(7). | Excluded (title and abstract review) |
| 367 | 徐树明, 杨晓棠, 王艳艳, 王生杰, 郑芳, 王秀云. 三维容积插值屏气检查序列在乳房肿瘤磁共振成像诊断中的应用. 肿瘤研究与临床. 2012;24(4). | Excluded (title and abstract review) |
| 368 | 许萍, 杨晓燕, 谢凡, 任安平, 穆伟斌, 桑鋆智. 磁共振动态增强对乳腺癌的早期诊断及临床应用价值. 产业与科技论坛. 2017;16(7):62-3. | Excluded (title and abstract review) |
| 369 | 闫少华, 牛永超, 彭保成, 马园, 李振玉. 探讨基于动态增强MRI和扩散加权成像的影像组学模型对小乳腺癌的诊断效果评价. 黑龙江医学. 2022;46(07):829-32. | Excluded (title and abstract review) |
| 370 | 杨化云, 刘英刚, 于逍, 郑先见. 核磁共振联合彩超对乳腺癌淋巴结转移的诊断效果. 影像研究与医学应用. 2018(23). | Excluded (title and abstract review) |
| 371 | 杨乐, 骆松芝, 史立强, 崔国金. 18F-FDGPET/CT在诊断浸润性乳腺癌和淋巴结转移的临床意义. 中医学报. 2014(B12). | Excluded (title and abstract review) |
| 372 | 杨文杰. 乳腺MRI与病理MP分级在乳腺癌新辅助化疗疗效评估临床应用中的相关性分析 2022. | Excluded (title and abstract review) |
| 373 | 杨文强, 佘青, 唐铁雷, 刘敏. 动态增强MRI对女性乳腺疾病性质的鉴别诊断价值研究. 中国CT和MRI杂志. 2021;19(11):99-101. | Excluded (title and abstract review) |
| 374 | 尹洪磊, 刘奇伦, 李娟. 核磁共振检查评价乳腺癌行保乳手术的可行性分析. 宁夏医科大学学报. 2009;31(3). | Excluded (title and abstract review) |
| 375 | 尹洪磊. 乳腺癌MRI临床应用研究 2009. | Excluded (title and abstract review) |
| 376 | 于芷晗, 王亮亮, 赵雪峰. 动态增强核磁共振在早期乳腺癌保乳手术中的应用研究. 组织工程与重建外科杂志. 2018;14(6). | Excluded (title and abstract review) |
| 377 | 袁才雅. 数字乳腺摄影与磁共振诊断乳腺癌的临床价值. 现代医学与健康研究电子杂志. 2021;5(15):111-3. | Excluded (title and abstract review) |
| 378 | 原晓燕. 循环肿瘤细胞及循环肿瘤DNA在乳腺癌中的临床应用研究 2016. | Excluded (title and abstract review) |
| 379 | 岳叶洼团, 熊春来. 磁共振T2WI序列瘤周水肿征象用于润性乳腺癌早期复发诊断的价值分析. 影像研究与医学应用. 2021;5(21):44-6. | Excluded (title and abstract review) |
| 380 | 张斌, 孟然, 赵洪猛, 曹旭晨. 隐匿型乳腺癌的临床诊治要点. 中国肿瘤临床. 2010;37(8). | Excluded (title and abstract review) |
| 381 | 张洪涛, 俞鸿凯, 王海屹, 叶慧义. 影像组学在前列腺癌中的应用进展. 微创泌尿外科杂志. 2018;7(04):282-6. | Excluded (title and abstract review) |
| 382 | 张继文, 贾红燕. 影像技术在预测乳腺癌新辅助化疗疗效中的应用进展. 临床肿瘤学杂志. 2023;28(09):849-55. | Excluded (title and abstract review) |
| 383 | 张杰. 新疆肿瘤医院2001-2010年可手术女性乳腺癌患者临床病理特征及诊疗模式的演变：一项单中心研究 2016. | Excluded (title and abstract review) |
| 384 | 张进, 庄琰, 杜森, 任文妍, 杨洁. MR淋巴管造影联合DWI序列诊断乳腺癌前哨淋巴结转移的临床分析. 山东医学高等专科学校学报. 2021;43(1). | Excluded (title and abstract review) |
| 385 | 张静静. 近红外响应性原位疫苗用于放大光热消融诱导的抗肿瘤免疫反应 2021. | Excluded (title and abstract review) |
| 386 | 张俊杰, 崔艳芬, 杨晓棠, 苗燕, 张婷, 杨钊. 术前乳腺MRI联合腋窝超声预测乳腺浸润性导管癌脉管侵犯. 中华放射学杂志. 2023;57(01):60-6. | Excluded (title and abstract review) |
| 387 | 张璐, 吴松. 影像基因组学及其在肾透明细胞癌中的研究进展. 肿瘤防治研究. 2019;46(05):486-9. | Excluded (title and abstract review) |
| 388 | 张倩, 俱京涛, 张雅琼, 张春谦. 磁共振动态增强结合血循环肿瘤细胞、循环游离DNA对乳腺癌的临床诊断研究. 安徽医药. 2024;28(4):732-6,后插3. | Excluded (title and abstract review) |
| 389 | 张清华, 李海涛, 方国旭, 郭鹏飞, 刘景丰. 深度学习在原发性肝癌相关诊断模型中的应用与前景. 临床肝胆病杂志. 2022;38(01):20-5. | Excluded (title and abstract review) |
| 390 | 张清泉, 王世军. 宫颈癌淋巴结的评估与手术策略探讨. 中国全科医学. 2022;25(18):2235-8. | Excluded (title and abstract review) |
| 391 | 张荣魁. 3.0T核磁检查在乳腺癌与腋窝淋巴结转移的相关性研究 2012. | Excluded (title and abstract review) |
| 392 | 张薇. 缺氧标记物CAIX表达与乳腺癌耐药性及侵袭转移能力间关系的研究 2013. | Excluded (title and abstract review) |
| 393 | 张兴梅, 王铭扬, 常占平, 张刚. 乳腺浸润性癌MRI表现与生物因子表达的相关性研究. 癌症进展. 2017;15(3). | Excluded (title and abstract review) |
| 394 | 赵闯, 戴朝六. 提高壶腹周围癌术前定性诊断的方法及意义. 肝胆外科杂志. 2018;26(04):245-50. | Excluded (title and abstract review) |
| 395 | 赵继红, 丁军, 徐松柏, 王显荣, 付卫光. ^18F-FDG PET/CT与MRI对乳腺癌诊断价值的比较. 吉林大学学报·医学版. 2011;37(4). | Excluded (title and abstract review) |
| 396 | 赵瑾, 黄林平. 早期乳腺癌的射频消融治疗. 中日友好医院学报. 2011;25(5). | Excluded (title and abstract review) |
| 397 | 赵儒钢. 脑转移瘤伴难治性瘤周水肿的治疗策略研究 2016. | Excluded (title and abstract review) |
| 398 | 赵爽, 魏国辉, 马志庆, 赵文华. 基于定量影像组学的乳腺肿瘤良恶性诊断. 中国生物医学工程学报. 2019;38(05):549-57. | Excluded (title and abstract review) |
| 399 | 郑春辉. 乳腺癌新辅助化疗后退缩模式与肿瘤异质性的相关性研究 2021. | Excluded (title and abstract review) |
| 400 | 郑新宇, 马帅. 隐匿性乳腺癌诊断与治疗. 中国实用外科杂志. 2013;33(3). | Excluded (title and abstract review) |
| 401 | 周涵. 多参数MRI联合临床特征提高BI-RADS 4类乳腺病变的恶性预测能力 2022. | Excluded (title and abstract review) |
| 402 | 周玲燕. 磁共振及乳腺钼靶应用于早期乳腺癌的影像学诊断效能对比. 影像研究与医学应用. 2023;7(23). | Excluded (title and abstract review) |
| 403 | 周学依. 基于钆造影剂的核磁共振成像检测乳腺癌腋窝淋巴结. 自我保健. 2022(7):122-4. | Excluded (title and abstract review) |
| 404 | 朱娟, 朱宜春. MRI乳腺背景实质强化与乳腺癌分子亚型的相关性研究. 河北医学. 2019;25(9). | Excluded (title and abstract review) |
| 405 | 朱学慧, 荣超. 人工智能技术在宫颈癌筛查和精准临床诊疗中的研究进展. 重庆医科大学学报. 2023;48(12):1477-82. | Excluded (title and abstract review) |
| 406 | 庄晓生. 多模态磁共振影像组学预测乳腺肿瘤退缩模式价值探索 2021. | Excluded (title and abstract review) |
| 407 | 庄晓曌, 李建军, 符莉莉, 蔡亲磊. 扩散加权成像对直肠癌患者根治术后局部复发的诊断价值. 中国医药导报. 2019;16(16):129-32+37. | Excluded (title and abstract review) |
