# Supplementary material for: Diagnostic performance of DCE-MRI radiomics in predicting axillary lymph node metastasis in breast cancer patients: A meta-analysis
Source: PLoS One. 2024 Dec 3;19(12):e0314653. doi: 10.1371/journal.pone.0314653 (PMC11614294; doi:10.1371/journal.pone.0314653)
Supplement: S4 Table — (DOCX) [file pone.0314653.s005.docx]

S4 Table. Results of sensitivity analysis.

| Eliminated study | Sensitivity | Specificity | PLR | NLR | DOR | AUC |
| --- | --- | --- | --- | --- | --- | --- |
| Arefan D, 2020 | 0.81 (0.77, 0.84) | 0.85 (0.81, 0.88) | 5.31 (4.35, 6.49) | 0.23 (0.19, 0.27) | 23.52 (17.24, 32.09) | 0.90 (0.87, 0.92) |
| Chen DX, 2022 | 0.81 (0.77, 0.84) | 0.84 (0.81, 0.86) | 4.97 (4.18, 5.91) | 0.23 (0.19, 0.28) | 21.70 (16.35, 28.80) | 0.89 (0.86, 0.92) |
| Chen JM, 2021 | 0.81 (0.77, 0.84) | 0.84 (0.81, 0.87) | 5.05 (4.20, 6.07) | 0.23 (0.19, 0.27) | 22.39 (16.60, 30.22) | 0.89 (0.86, 0.92) |
| Chen WY, 2024 | 0.81 (0.77, 0.84) | 0.85 (0.81, 0.88) | 5.31 (4.34, 6.49) | 0.22 (0.19, 0.27) | 23.61 (17.26, 32.30) | 0.90 (0.87, 0.92) |
| Cheng Y, 2022 | 0.81 (0.78, 0.85) | 0.85 (0.81, 0.87) | 5.27 (4.32, 6.44) | 0.22 (0.18, 0.26) | 24.03 (17.75, 32.52) | 0.90 (0.87, 0.92) |
| Cui XY, 2019 | 0.80 (0.77, 0.83) | 0.85 (0.81, 0.88) | 5.26 (4.30, 6.43) | 0.23 (0.20, 0.28) | 22.51 (16.65, 30.43) | 0.89 (0.86, 0.92) |
| Han L, 2019 | 0.81 (0.78, 0.85) | 0.84 (0.81, 0.87) | 5.17 (4.24, 6.31) | 0.22 (0.18, 0.27) | 23.45 (17.15, 32.08) | 0.90 (0.87, 0.92) |
| Liu CL, 2019 | 0.81 (0.77, 0.84) | 0.85 (0.81, 0.87) | 5.24 (4.28, 6.40) | 0.23 (0.19, 0.27) | 23.04 (16.90, 31.40) | 0.90 (0.87, 0.92) |
| Liu J, 2019 | 0.81 (0.77, 0.84) | 0.85 (0.81, 0.87) | 5.27 (4.33, 6.42) | 0.22 (0.19, 0.27) | 23.61 (17.38, 32.07) | 0.90 (0.87, 0.92) |
| Liu Y, 2022 | 0.80 (0.76, 0.83) | 0.85 (0.83, 0.88) | 5.45 (4.53, 6.55) | 0.23 (0.20, 0.28) | 23.20 (17.06, 31.55) | 0.90 (0.87, 0.92) |
| Ma MM, 2022 | 0.81 (0.77, 0.84) | 0.85 (0.81, 0.87) | 5.22 (4.29, 6.36) | 0.23 (0.19, 0.27) | 22.96 (16.96, 31.07) | 0.90 (0.87, 0.92) |
| Santucci D, 2021 | 0.81 (0.77, 0.84) | 0.85 (0.82, 0.88) | 5.31 (4.36, 6.47) | 0.23 (0.19, 0.27) | 23.28 (17.11, 31.68) | 0.90 (0.87, 0.92) |
| Shan YN, 2020 | 0.81 (0.77, 0.84) | 0.85 (0.82, 0.88) | 5.34 (4.38, 6.51) | 0.22 (0.19, 0.27) | 23.74 (17.44, 32.34) | 0.90 (0.87, 0.92) |
| Song DL, 2022 | 0.81 (0.77, 0.84) | 0.85 (0.81, 0.88) | 5.28 (4.31, 6.46) | 0.22 (0.19, 0.27) | 23.50 (17.17, 32.18) | 0.90 (0.87, 0.92) |
| Tang YQ, 2022 | 0.80 (0.76, 0.83) | 0.84 (0.81, 0.87) | 5.08 (4.19, 6.15) | 0.23 (0.20, 0.28) | 21.60 (16.27, 28.68) | 0.89 (0.86, 0.92) |
| Wang CH, 2021 | 0.81 (0.77, 0.85) | 0.85 (0.82, 0.88) | 5.40 (4.47, 6.54) | 0.22 (0.18, 0.27) | 24.44 (18.25, 32.72) | 0.90 (0.87, 0.92) |
| Wang Q, 2024 | 0.81 (0.77, 0.85) | 0.85 (0.81, 0.88) | 5.29 (4.32, 6.47) | 0.22 (0.18, 0.27) | 23.85 (17.47, 32.56) | 0.90 (0.87, 0.92) |
| Wang YX, 2024 | 0.81 (0.77, 0.85) | 0.85 (0.81, 0.88) | 5.34 (4.38, 6.52) | 0.22 (0.18, 0.27) | 24.12 (17.77, 32.74) | 0.90 (0.87, 0.92) |
| Zhan CA, 2021 | 0.80 (0.76, 0.83) | 0.84 (0.81, 0.87) | 5.11 (4.21, 6.20) | 0.24 (0.20, 0.28) | 21.64 (16.32, 28.69) | 0.89 (0.86, 0.91) |
| Zhang CM, 2023 | 0.81 (0.77, 0.85) | 0.85 (0.81, 0.87) | 5.27 (4.31, 6.44) | 0.22 (0.18, 0.27) | 23.68 (17.35, 32.32) | 0.90 (0.87, 0.92) |
| Zhang JW, 2023 | 0.81 (0.77, 0.84) | 0.85 (0.81, 0.88) | 5.29 (4.33, 6.47) | 0.22 (0.18, 0.27) | 23.69 (17.35, 32.36) | 0.90 (0.87, 0.92) |
| Zhao NN, 2023 | 0.80 (0.77, 0.84) | 0.84 (0.81, 0.87) | 5.06 (4.20, 6.09) | 0.23 (0.19, 0.28) | 21.79 (16.39, 28.98) | 0.89 (0.86, 0.92) |
| Zhu YD, 2021 | 0.81 (0.77, 0.85) | 0.84 (0.81, 0.87) | 5.16 (4.24, 6.28) | 0.22 (0.18, 0.27) | 23.22 (17.01, 31.70) | 0.90 (0.87, 0.92) |
| Zhu YQ, 2022 | 0.81 (0.78, 0.85) | 0.84 (0.81, 0.87) | 5.23 (4.28, 6.39) | 0.22 (0.18, 0.27) | 23.60 (17.31, 32.19) | 0.90 (0.87, 0.92) |

PLR: positive likelihood ratio; NLR: negative likelihood ratio; DOR: diagnostic odds ratio; AUC: area under the curve.
